# Eco-Friendly Cellulose-Supported Nickel Complex as an Efficient and Recyclable Heterogeneous Catalyst for Suzuki Cross-Coupling Reaction

**DOI:** 10.3390/molecules29194525

**Published:** 2024-09-24

**Authors:** Zhanyu Li, Guohao Zhou, Yu Sun, Yingning Mao, Fanxiang Zeng, Zhihui Wang, Yuanyuan Zhang, Bin Li

**Affiliations:** 1 College of Chemistry Chemical Engineering and Resource Utilization, Northeast Forestry University, Harbin 150040, China; lizy567@nefu.edu.cn (Z.L.); 2021115434@nefu.edu.cn (G.Z.); 15161125726@163.com (Y.M.); zfx5192022@163.com (F.Z.); 18147574140@163.com (Z.W.); zhangyuanyuan@nefu.edu.cn (Y.Z.); 2 Post-Doctoral Mobile Research Station of Forestry Engineering, Northeast Forestry University, Harbin 150040, China; 3 Heilongjiang Ecological Engineering College, Harbin 150025, China; wjl33@nefu.edu.cn

**Keywords:** cellulose, heterogeneous catalyst, Suzuki cross-coupling reaction, nickel complex

## Abstract

In this work, we applied commercially available 2-pyridinecarboxylic acid to modify cellulose by simple manipulations, and then anchored low-toxicity metal nickel onto the modified cellulose to prepare the heterogeneous catalyst (CL-AcPy-Ni). The obtained catalyst was characterized by FT-IR, TG-DSC, BET, XRD, SEM-EDS, ICP-OES, XPS, and GPC. The catalytic performance of CL-AcPy-Ni in the Suzuki cross-coupling reaction was investigated using 4-methyl iodobenzene and phenylboronic acid as the model substrates reacting in THF under 120 °C for 24 h. The catalytic ability of CL-AcPy-Ni for various halobenzenes and phenylboronic acid derivatives was also further investigated under optimal conditions and demonstrated good catalytic activity, and a series of diaryls were successfully synthesized. Finally, this green nickel-based catalyst could be reused for five successive cycles by simple centrifugation.

## 1. Introduction

Transition metal-catalyzed cross-coupling reactions have been widely used in the synthesis of bioactive molecules [1], compounds for agriculture [2], and materials [3]. Of these powerful reactions, the palladium-catalyzed Suzuki cross-coupling reaction has received a great deal of attention since its discovery in 1979 [4]. Due to its excellent functional group tolerance, high reactivity, and mild reaction conditions, the Suzuki cross-coupling reaction enabled the efficient synthesis of alkanes [5,6], olefins [7,8], biphenyl skeletons, etc. [9,10,11]. The development of the Suzuki cross-coupling reaction has contributed greatly to organic synthetic chemistry; however, the reutilization of the homogeneous catalyst palladium has turned out to be a shortcoming of the coupling reaction. With the development of heterogeneous catalysis, massive progress has been made by means of anchoring a metal on solid supports, which would make the precious metal palladium recyclable [12,13,14]. Various new types of supports have been introduced by researchers, such as MOFs [15], chitin [16], oxide [17,18], chitosan [19,20,21], and resin [22]. Recently, with the ever-increasing awareness of environmental protection and sustainable development, the applications of biodegradable, renewable, and non-toxic natural polymers have gained increasing attention [23,24].

Cellulose is the most abundant natural polymer, and its high thermal stability, easy modification, and excellent metal-binding ability make it an attractive candidate as a support for anchoring metal catalysts [25,26]. Numerous superior modified cellulose-immobilized metal catalysts efficiently promote the Suzuki cross-coupling reaction. Among these profiles, initial investigations loaded metals directly onto cellulose [27,28,29,30,31,32]. However, the metal catalysts readily leached owing to the weak coordination of the hydroxyl groups [33]. Subsequent approaches combined cellulose with inorganic [34,35] or polymeric materials [36,37,38,39,40] and then immobilized the metals. These approaches effectively solved the problem of metal dissociation during the catalytic processes, but the catalytic properties of metals cannot be tamed. To better improve the performance of cellulose-based heterogeneous catalysts, various organic molecular ligands were grafted onto the cellulose to improve the catalytic behavior, for instance, *N*-Heterocyclic carbene [41,42,43], organophosphine [44], and nitrogen-containing ligands [45,46,47,48,49]. Although the introduction of organic ligands could both effectively immobilize metal and improve metal catalytic activity, the following problems still remain: (1) tedious synthesis steps, and (2) the use of toxic, costly metals.

Standing on the existing research landscape, we aimed to fix cellulose with simple organic molecules to simplify the cellulose modification steps. More importantly, we used green, low-toxicity metal nickel instead of highly toxic precious metals. We selected the commercially available 2-pyridinecarboxylic acid as the synthetic block to modify cellulose, and then immobilized the transition metal nickel, finally leading to a green heterogeneous nickel catalyst (CL-AcPy-Ni). The CL-AcPy-Ni was characterized by FT-IR, XRD, XPS, SEM-EDS, ICP-OES, TG-DSC, and GPC. With this cellulose-supported nickel catalyst in hand, the catalytic performance in Suzuki cross-coupling was investigated using 4-iodotoluene and phenylboronic acid as model substrates, obtaining the coupling product in good yield. Next, we also explored the generality of the substrates, which had excellent functional group tolerance to various types of arylhalides and arylboronic acids. Finally, the heterogeneous catalyst was readily reused by simple centrifugal separation in successive runs. This work pioneered a greener cellulose-supported heterogeneous catalyst, which offers a distinctive insight for the further application of biopolymers.

## 2. Results and Discussion

### 2.1. Catalyst Characterization

**Figure 1:**
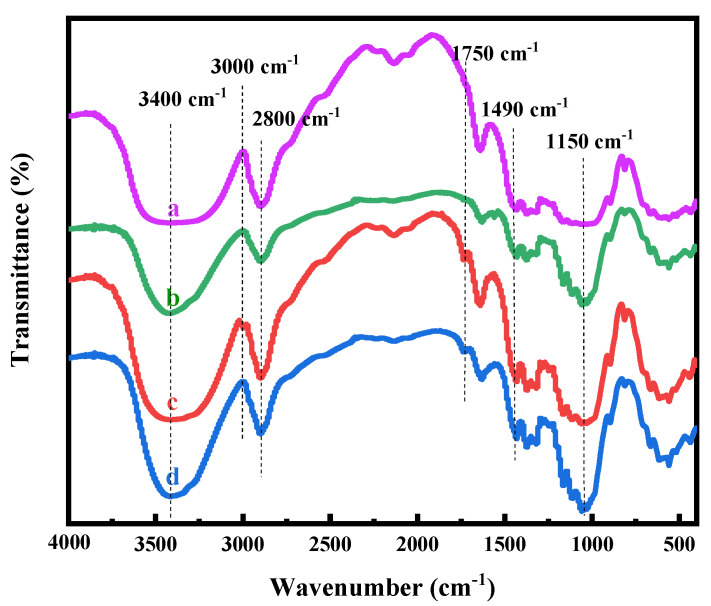
Infrared spectrum of a cellulose; b acid-activated cellulose; c 2-pyridine-modified cellulose; d CL-AcPy-Ni.

Initially, the FT-IR spectra were used to characterize the 2-pyridinecarboxylic acid-modified microcrystalline cellulose, and the infrared spectra are shown in Figure 1. Spectrum a was the spectrum of microcrystalline cellulose, in which the broad peak at 3400 cm^−1^ belonged to the hydroxyl group peak, 2800 cm^−1^ corresponded to the stretching vibration of the C-H bond, 1490 cm^−1^ was related to the in-plane bending vibration of the C-H bond, and 1150 cm^−1^ belonged to the stretching vibration of the C-C bond [50]. Infrared spectrum b was the sulfuric acid-activated cellulose, and there was no significant change compared to infrared spectrum a, indicating that the functional groups of the cellulose were not changed. Spectrum c was the 2-pyridinecarboxylic acid-modified cellulose (CL-AcPy), and a new peak appeared at 3000 cm^−1^ belonging to the C-H stretching vibration of the pyridine ring, and the characteristic peak at 1750 cm^−1^ corresponded to the ester groups. Spectrum d was the CL-AcPy-Ni; the characteristic peak at 3000 cm^−1^ became insignificant, which may be due to the coordination of the metal nickel [51,52,53].

**Figure 2:**
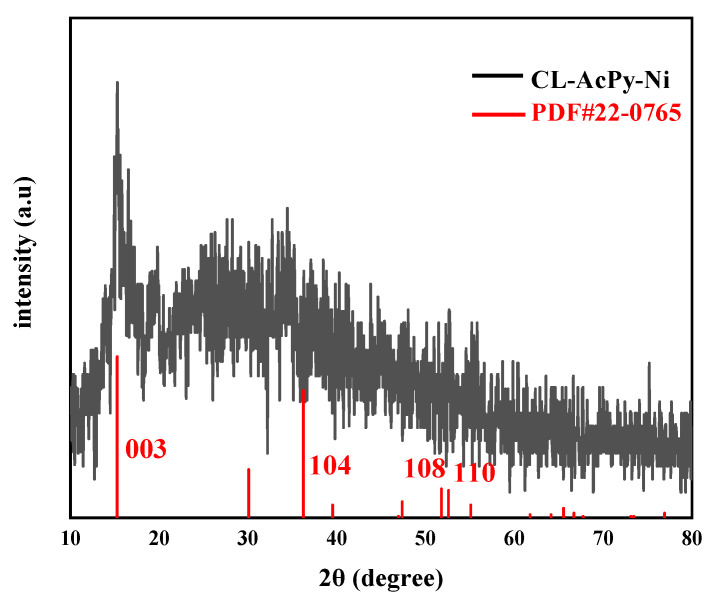
XRD pattern of CL-AcPy-Ni.

The crystal structure of the catalyst was investigated using XRD as shown in Figure 2. The 2θ degrees = 15.260°, 36.240°, 51.780°, and 52.580° in the catalyst, which correspond to the cubic crystal planes (003), (104), (018), and (110) of nickel chloride, respectively, are significantly weakened in their intensity when compared to the standard diagram of NiCl_2_ (PDF #22-0765). This further indicates that the preparation of cellulose-supported catalyst was successful.

**Figure 3:**
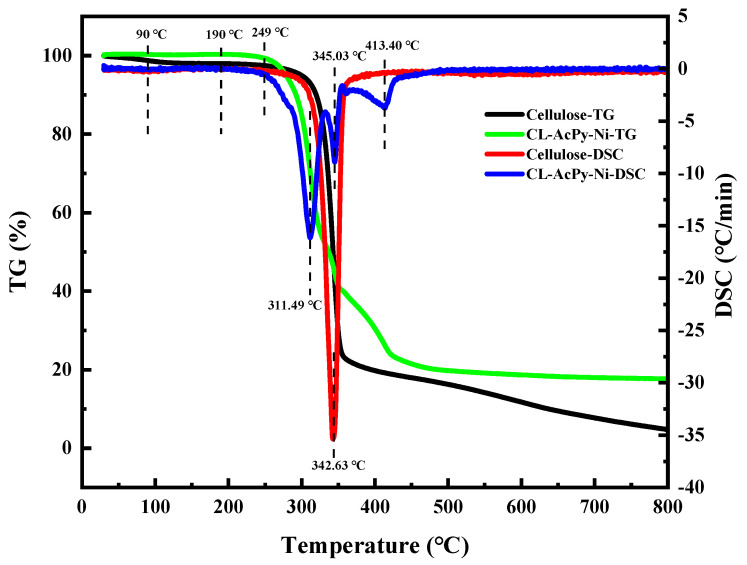
TG−DSC analysis of cellulose and CL-AcPy-Ni.

In order to evaluate the stability of cellulose and CL-AcPy-Ni, TG experiments were performed and the results are presented in Figure 3. There were four main pyrolysis regions for the cellulose. There was a slight mass loss of cellulose between 50 °C and 90 °C, which could be attributed to the loss of free water. The temperature between 90 °C and 190 °C might be responsible for the evaporation of bound water in cellulose, resulting in a mass loss of 1.69 wt%. There was a dramatic mass decay from 300 °C to 400 °C, which was probably caused by the pyrolysis of cellulose at high temperatures to produce volatiles; at the same time, the maximum weight loss of cellulose occurred at 342.63 °C. At the stage of 400 °C to 800 °C, the cellulose backbone may have begun to collapse. The CL-AcPy-Ni TG experimental result showed a smaller mass loss of 0.49% from 50 °C to 190 °C; this result may be due to the removal of water during the cellulose modification process. The CL-AcPy-Ni started to decompose at the temperature of 249 °C, which was earlier than the cellulose decomposition temperature. It may be caused by the coordination effect of the metal with the cellulose that activates the chemical bonds of the cellulose, making it less stable. The first decomposition range of CL-AcPy-Ni was 249 to 345.03 °C; there was a rapid mass decrease of 48.4 wt% between 249 °C and 311.49 °C. Another considerable weight loss temperature region was 345.03-500 °C; the breakage of the ester groups and the dehydration of aliphatic hydroxyl groups probably explain this. Between 500 and 800 °C, the main reason for mass loss may be the cleavage of the cellulose skeleton. The TG data demonstrated the excellent thermal stability of the CL-AcPy-Ni catalyst, with a minimum decomposition temperature of 249 °C, which provided a robust foundation for efficiently catalyzing Suzuki cross-coupling reactions [54,55].

**Figure 4:**
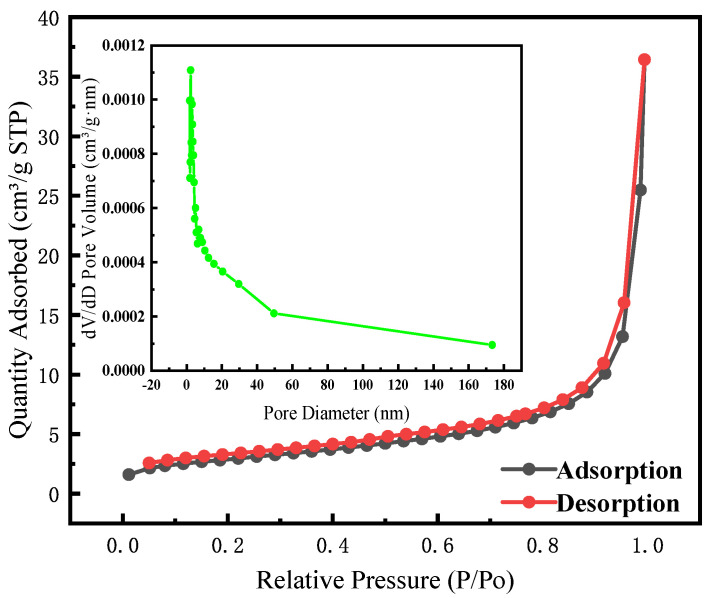
Nitrogen adsorption/desorption isotherms of CL-AcPy-Ni.

According to IUPAC, the N_2_ adsorption–desorption isotherms of the CL-AcPy-Ni complex belonged to the type II adsorption isotherm, as shown in Figure 4. From this, we calculated the specific surface area and the average pore size of the CL-AcPy-Ni: the specific surface area was 10.4336 m^2^/g, and the average pore size was 22.0587 nm [56,57,58,59]. The results of the pore diameter test showed that the pore sizes were distributed around 10 nm (see green curve).

**Figure 5:**
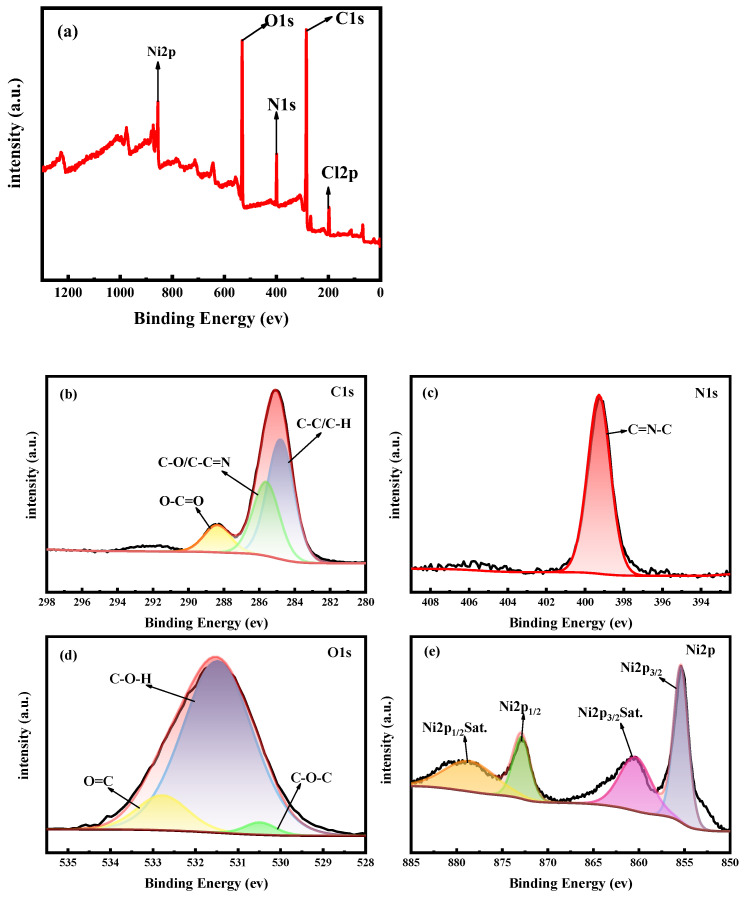
XPS spectra of CL-AcPy-Ni in the regions of (**a**) survey; (**b**) C 1s; (**c**) N 1s; (**d**) O 1s; (**e**) Ni 2p.

Next, to learn more about the properties of the catalyst, the XPS was used to characterize the oxidation state of the elements and the surface elemental formulation, which could provide evidence for the introduction of pyridine and metal nickel (Figure 5). In spectrum (a), the C, Cl, O, N, and Ni could be found in CL-AcPy-Ni; these clues provided straightforward evidence of the successful preparation of the catalyst. The spectrum of C1s in spectrum (b) contained three sub-peaks with a binding energy of 284.8 eV, derived from the C-C and C-H bonds, and 288.38 eV corresponding to the C=O bonds of ester, with C=N leading to 285.61 eV. In spectrum (c), a typical peak was located at 399.28 eV, which could be the N in the pyridine. There were three typical peaks in spectrum (d), including two broad and low peaks observed at 530.48 eV and 532.78 eV, corresponding to C-O-C and C=O bonds for ester groups, respectively. A high and broad peak at 531.48 eV was presumed to be C-O-H. Spectrum (e) was the XPS spectra of Ni2p, in which the binding energies of Ni2p_3/2_ and Ni2p_1/2_ were 855.38 eV and 872.88 eV, respectively. The binding energies of the satellite peaks were 860.38 eV and 878.58 eV, respectively. Compared with the standard spectrum, the binding energies of C, O, and N in the CL-AcPy-Ni were all larger than the standard spectrum, which probably originated from the heteroatoms O and N coordinated to nickel (II), resulting in the transfer of electrons and affecting the density of the electron cloud, increasing the binding energy [60,61,62,63,64,65,66].

**Figure 6:**
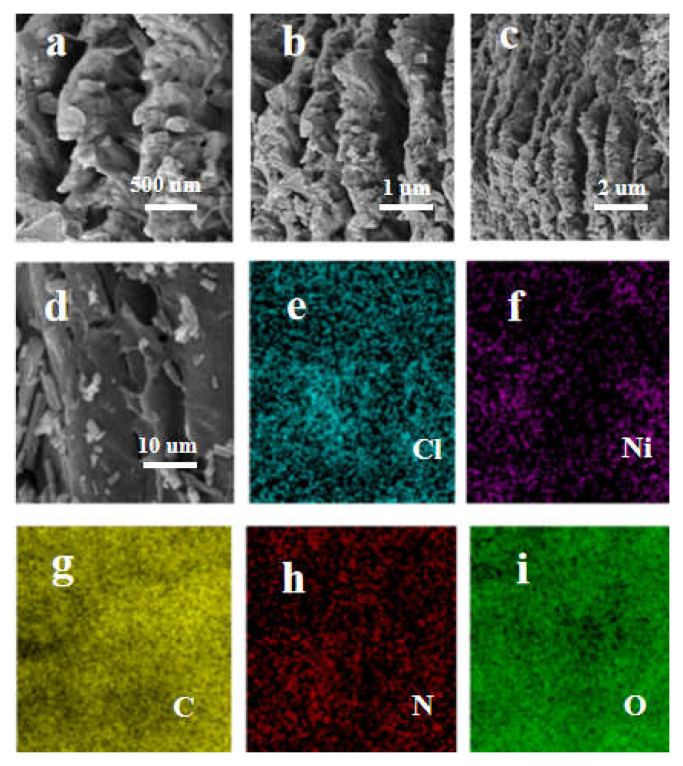
(**a**–**d**) Typical SEM image and (**e**–**i**) elemental EDS mapping image of CL-AcPy-Ni.

In order to identify the surface morphology and microstructure of the CL-AcPy-Ni more clearly and accurately, SEM was used to examine images of different sizes, as shown in Figure 6. The surface of the CL-AcPy-Ni image was an irregularly layered shape, and the nickel nanoparticles were uniformly anchored. Finally, the EDS spectrum clearly showed the presence of Cl, N, O, and C atoms (e, g, h, i). At the same time, the uniform distribution of nickel observed in the EDS spectrum further indicated the successful preparation of the heterogeneous CL-AcPy-Ni catalyst.

### 2.2. CL-AcPy-Ni Catalytic Performance in Suzuki Cross-Coupling

With the prepared heterogeneous CL-AcPy-Ni catalyst in hand, the 4-methyliodobenzene and phenylboronic acid were selected as model substrates to verify the catalytic activity of CL-AcPy-Ni (Table 1). Firstly, the reaction was explored with the base species using tetrahydrofuran as a solvent; when triethylamine was introduced as the base, the reaction did not occur (Entry 1). Next, we turned our attention to inorganic bases; the reaction was carried out with the strong inorganic bases NaOH and KOH, and the reaction occurred smoothly, but the yield was not satisfactory (Entries 2, 3). We speculated that strong basicity hydrolyzed the ester groups, leading to catalyst breakdown. Our speculation was confirmed by observing that the color of the catalyst changed from dark gray to white. Thus, weakly basic carbonates and potassium phosphate were mentioned as bases, with the carbonates giving a poor yield and the potassium phosphate giving the target product in moderate yield (Entries 4–6). Potassium phosphate was elected as the optimal base to evaluate the reaction conditions. Increasing the temperature from 100 °C to 120 °C had a positive effect on the conversion, delivering a respectable yield of 78% (Entry 7). The amount of base was reduced to 2.0 equivalents, reducing the yield to 50% (Entry 8). Finally, the effect of the polarity of the solvent on the reaction was investigated; the ether solvent dioxane gave a similar result to THF with a yield of 73%. Poor results were obtained for both non-polar solvent toluene and highly polar protonated solvent H_2_O (Entries 9–11).

After obtaining the optimal reaction conditions, the CL-APy-Ni-catalyzed Suzuki cross-coupling reactions of arylboronic acids and various arylhalides were examined (Table 2). The different types of arylhalides had a significant effect on the reaction: the highly reactive iodobenzene gave the highest yield of 74%, the less reactive bromobenzene obtained a poorer yield of 44%, and the inert chlorobenzene did not work (Table 2, **3a_1_**–**3a_3_**). With these results in hand, the reactive properties of various substituted iodobenzenes were investigated. For iodobenzenes bearing electron-withdrawing and electron-donating groups at the para-position, all were suitable materials (Table 2, **3b**, **3d**–**3h**). However, the electroneutral *p*-phenyliodobenzene achieved a 29% yield (Table 2, **3i**). This heterogeneous catalyst also showed excellent catalytic reactivity for *m*-substituted and di-substituted substrates (Table 2, **3c**, **3j**–**3k**). Nevertheless, the response to *o*-substituted iodobenzene was poor, which may be caused by the effect of bulky steric hindrance (Table 2, **3l**). In addition, the fused ring 1-iodonaphthalene could also respond smoothly, and the desired product was obtained in moderate yield (Table 2, **3m**).

Next, we investigated the functional group compatibility of arylboronic acids; Suzuki cross-coupling could take place smoothly for electron-withdrawing and electron-releasing group-modified arylboronic acids. It is worth noting that the reactivity of electron-withdrawing group-modified arylboronic acids showed a higher reactivity, probably because the electron-withdrawing arylboronic acids had a faster transmetallation rate (Table 2, **3n**–**3r**). For arylboronic acids decorated with electron-acceptor groups at the meta-position, corresponding biphenyl was obtained with a sacrifice in yield (Table 2, **3s**–**3t**). For multi-substituted arylboronic acids, cross-coupling was also possible, but with poor yield (Table 2, **3u**–**3v**). To our surprise, the catalyst still had good catalytic activity for 2-naphthaleneboronic acid, yielding the final product in moderate yield (Table 2, **3w**). The general procedure, characterization data and ^1^H NMR and ^13^C NMR spectra of **3a**–**w** can be found in the Supplementary Materials.

In recent years, heterogeneous nickel-catalyzed Suzuki cross-coupling reactions have also gained several valuable scientific results [67,68,69,70,71]. These approaches mainly focused on loading nickel onto inorganic materials, or a small complex organic molecule-modified inorganic supporter. Despite high reaction efficiencies, the modification of the catalyst’s structural diversity has major limitations. In addition, degradability should be further improved. Our pioneering approach achieved similar results; however, the green, abundant, biodegradable cellulose is more consonant with green chemistry.

### 2.3. Recyclability of Heterogeneous CL-AcPy-Ni Catalyst

**Figure 7:**
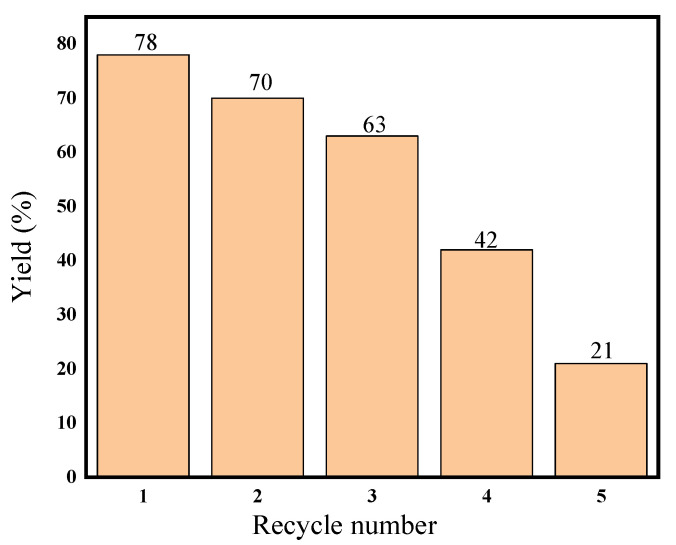
Recycling experiments of CL-AcPy-Ni in Suzuki reaction.

**Figure 8:**
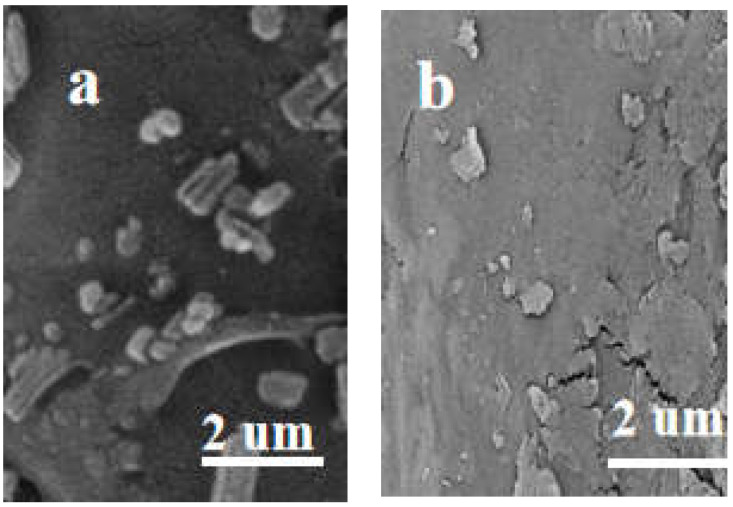
(**a**) Catalyst after 3 cycles; (**b**) catalyst after 5 cycles.

The cyclability of the catalyst was also investigated by catalyzing the cross-coupling of phenylboronic acid and iodobenzene (Figure 7). The catalyst maintained high catalytic performance after three cycles, but the catalytic activity decreased significantly in the fourth cycle. In order to find out the reason, we investigated the nickel metal loading in the catalyst using ICP-OES. According to the ICP-OES data, after five catalytic cycles, the metal content in the catalyst was obviously reduced, so the catalytic activity gradually decreased (Table 3). At the same time, the recovered catalyst was tested for SEM (see Figure 8a,b); as can be seen from the SEM images, the surface of the catalyst becomes smoother after five cycles. We speculated that this could be due to dissociation of the metal and hydrolysis of the ester groups.

### 2.4. Possible Mechanism of CL-AcPy-Ni-Catalyzed Suzuki Cross-Coupling

**Scheme 1:**
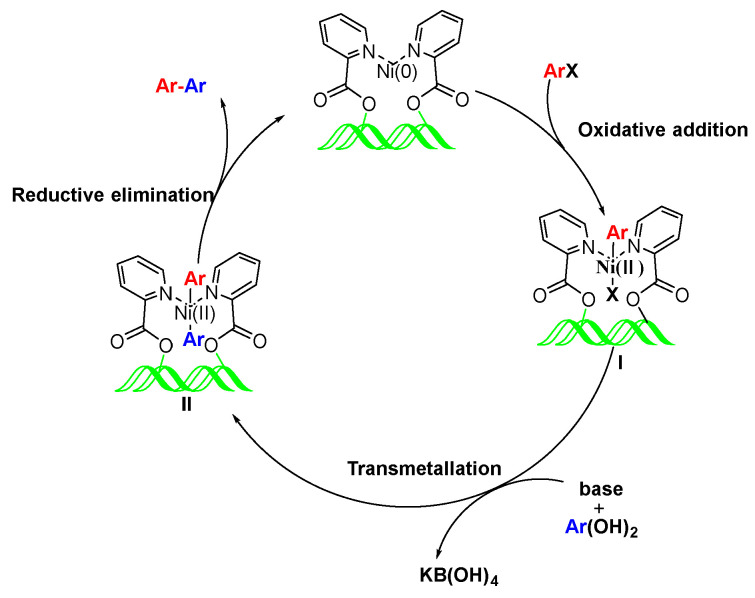
Possible reaction mechanism.

The reaction mechanism of the CL-AcPy-Ni-catalyzed cross-coupling is shown in Scheme 1. Firstly, Ni(II) was reduced by arylboronic acid to generate the reactive intermediate Ni(0), and then oxidative addition with arylhalide formed intermediate **I**, followed by transmetallation, which gave intermediate **II**; finally, intermediate **II** underwent reductive elimination to obtain the product, and the Ni(0) intermediate was regenerated for a new catalytic cycle.

Other material-loaded nickel catalysts have been reported, each with its own unique advantages. Compared with them, the catalytic effect of A is basically equal, but it has better catalytic activity for substrates containing fluorine-like groups, which is of great practical significance for future applications.

## 3. Materials and Methods

### 3.1. Materials

Microcrystalline cellulose was purchased from TCI (Shanghai, China) Chemical Industry Development Co., Ltd., Shanghai, China. Potassium phosphate anhydrous (K_3_PO_4_), potassium hydroxide (KOH), potassium methoxide (CH_3_OK), sodium sulfate (Na_2_SO_4_), arylhalides, and 2-pyridinecarboxylic acid were purchased from Shanghai Energy Co., Ltd., China (Shanghai, China). Other reagents and solvents, such as EtOH, DMF, THF, and ethyl acetate (EA), were supplied by Tianjin Fuyu Chemical Co., Ltd., China (Tianjin, China). Unless otherwise noted, all chemical reagents (AR grade) applied in this study were used as received without further purification.

### 3.2. General Remarks

The CL-AcPy-Ni and CL-AcPy were characterized by various methods. The instruments and test methods are listed as follows: the Fourier transform infrared spectroscopy (FT-IR) analysis of all the samples was performed on a Nicolet iS10 spectrometer (Thermo Fisher, Waltham, MA, USA). KBr was used as the solid dispersant and mixed with the sample in a ratio of 50:1 to 100:1. The mixed samples were scanned from 500 cm^−1^ to 4000 cm^−1^ at room temperature. TG-DSC (NETZSCH, DE) was used to evaluate the thermal stability of materials under a N_2_ atmosphere, and heated from 50 °C to 800 °C at a rate of 10 °C/min^−1^. Under the standard degassing conditions of a Micromeritics Instrument, the samples were pretreated under a vacuum at 100 °C for 4 h, and then nitrogen adsorption and desorption tests were carried out using BET analyzer (Micromeritics, Norcross, GA, USA). We used an inductively coupled plasma–optical emission spectrometer analyzer (ICP-OES) to determine the nickel content in the catalyst. The bonding mode of different atoms and the valence state of nickel were determined by XPS (Thermo Scientific K-Alpha, MA, USA). The surface morphology of the catalyst was examined by SEM (Tescan, Brno, Czech Republic). The crystal structure and crystalline state of the material were analyzed by powder X-ray diffraction (XRD) in the 2*θ* range of 10°–90°. The molecular weight of the sample was determined by gel permeation chromatography (PL GPC50) with a mobile phase of DMSO at a concentration of 0.1 (mg/mL) and an injection volume of 100 μL. The NMR spectra were recorded for ^1^H NMR and ^13^C NMR using TMS as an internal standard (Bruker, Billerica, MA, USA, AV 400). The chemical shifts were expressed in ppm and coupling constants in Hertz (Hz). Data for ^1^H NMR were recorded as follows: chemical shift (δ, ppm), splitting (s = singlet; d = doublet; dd = doublet of doublets; t = triplet; td = triplet of doublets; q = quartet; m = multiplet).

### 3.3. General Procedure for the Preparation of CL-AcPy-Ni

#### 3.3.1. Preparation of 2-Pyridinecarbonyl Chloride

A mixture of 2-pyridinecarboxylic acid (12.5 g) and SOCl_2_ (25 mL) was stirred at 65 °C for 4 h in a 50 mL round-bottom flask. At the end of the reaction, the mixture was cooled to room temperature and the solvent was removed to give a burgundy solid. The resulting solid was dried in an oven at 70 °C for 12 h, and then washed with toluene 2 to 3 times to obtain a gray or pink solid [72].

#### 3.3.2. Pre-Activation of Microcrystalline Cellulose

We placed 13.5 g microcrystalline cellulose into a 100 mL round-bottom flask, added 50 mL of 5% sulfuric acid solution, stirred it at 50 °C for 24 h, and then removed the solvent by filtration; the resulting solid was washed with distilled water to pH = 7, and then vacuum-dried at 55 °C.

#### 3.3.3. Preparation of CL-AcPy

**Scheme 2:**
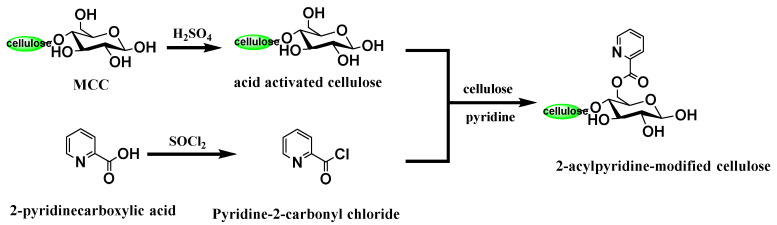
Preparation of 2-pyridine-modified cellulose.

A 100 mL round-bottom flask was charged with pre-activated microcrystalline cellulose (13.5 g) and 50 mL pyridine as a solvent; prepared 2-pyridinecarbonyl chloride (14.2 g) was slowly added, and then the mixture was stirred at 80 °C for 24 h (Scheme 2). At the end of the reaction, the mixture was cooled to room temperature and filtered to obtain a solid, which was washed with ethanol 6–8 times and then washed with acetone until the filtrate turned clear [73,74,75,76,77,78].

#### 3.3.4. Preparation of CL-AcPy-Ni

**Scheme 3:**
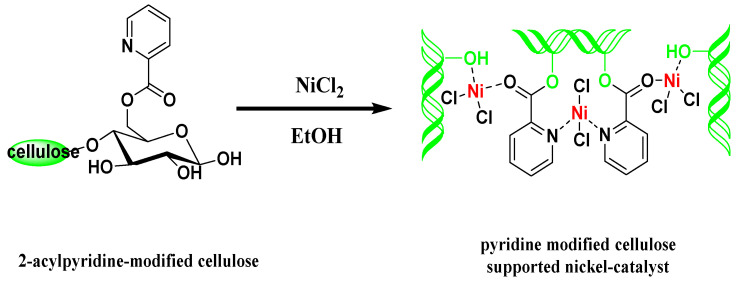
Synthesis of CL-AcPy-Ni.

Under a nitrogen atmosphere, anhydrous nickel chloride (13 g) and the obtained CL-AcPy (30.1 g) were added to 40 mL anhydrous ethanol, and the resultant mixture was stirred at 80 °C for 8 h (Scheme 3). The crude catalyst was obtained by filtration at the end of the reaction and then washed with ethanol 6–8 times, giving the CL-AcPy-Ni.

## 4. Conclusions

We successfully used commercially available 2-pyridinecarboxylic acid to modify cellulose and prepared an environmentally friendly heterogeneous catalyst CL-AcPy-Ni. The cellulose-based catalyst was characterized by FT-IR, TG-DSC, BET, XRD, SEM-EDS, ICP-OES, XPS, etc. This catalyst was easily accessible by simple manipulation and showed good catalytic activity. The CL-AcPy-Ni could well catalyze the cross-coupling reaction of arylboronic acids and arylhalides, and provided good functional group tolerance. Finally, the recyclability of the catalyst was further explored; this heterogeneous catalyst could be recycled by simple centrifugal separation at the end of the reaction, and showed good recoverability after three runs. The preparation of this environmentally friendly heterogeneous cellulose-based catalyst will provide new research ideas for subsequent studies.

## Figures and Tables

**Table 1:** The optimization of Suzuki cross-coupling.

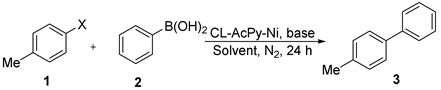				
**Entry ^a^**	**Base**	**Solvent**	**Temperature**	**Yield (%) ^b^**
1	Et_3_N	THF	100	none
2	NaOH	THF	100	24
3	KOH	THF	100	27
4	Cs_2_CO_3_	THF	100	trace
5	K_2_CO_3_	THF	100	8
6	K_3_PO_4_	THF	100	60
7	K_3_PO_4_	THF	120	78
8 ^c^	K_3_PO_4_	THF	120	50
9	K_3_PO_4_	1,4-dioxane	120	73
10	K_3_PO_4_	toluene	120	trace
11	K_3_PO_4_	H_2_O	120	trace

^a^ Unless otherwise noted, the reaction was carried out with **1** (0.20 mmol), **2** (1.3 equiv.), CL-AcPy-Ni (96 mg, 10 mol%), base (3 equiv.), and solvent (1.5 mL) at 120 °C for 24 h; ^b^ yield of the isolated product; ^c^ 2 equiv. of base.

**Table 2:** CL-AcPy-Ni-catalyzed Suzuki cross-coupling reactions.

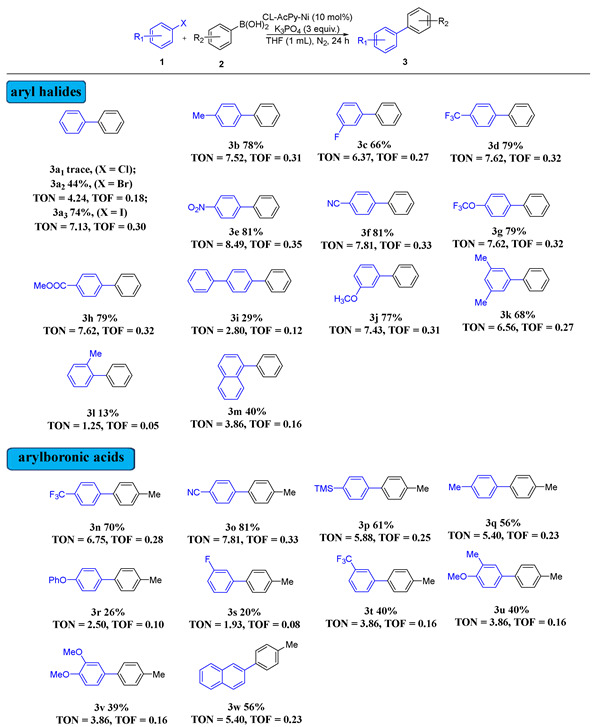

Reaction conditions: 1 (0.2 mmol), 2 (0.26 mmol), CL-AcPy-Ni (96 mg), K_3_PO_4_ (0.6 mmol), THF = (1 mL), N_2_, 120 °C, 24 h; Yield of the isolated product; TON = moles of product formed/moles of loaded nickel in the catalyst (based on ICP-OES); TOF = TON/time (h).

**Table 3:** The amount of nickel at each time of the cycle and leaching test.

Run	Fresh	1	2	3	4	5
Nickel content (mmol/g)	0.216	0.174	0.110	0.044	0.026	0.0108
Leaching ratio	78%	70	63%	42%	21%	-

## Data Availability

Data from the research described in the manuscript are available from the authors.

## Supplementary Materials

The following supporting information can be downloaded at https://www.mdpi.com/article/10.3390/molecules29194525/s1. 1. General procedure and characterization data for **3a**–**w**; 2. ^1^H NMR and ^13^C NMR spectra of **3a**–**w**. 3. Data of GPC.
