# Supplementary material for: Eco-Friendly Cellulose-Supported Nickel Complex as an Efficient and Recyclable Heterogeneous Catalyst for Suzuki Cross-Coupling Reaction
Source: Molecules. 2024 Sep 24;29(19):4525. doi: 10.3390/molecules29194525 (PMC11477910; doi:10.3390/molecules29194525)
Supplement: Supplementary file 1 [file molecules-29-04525-s001.zip › molecules-3180532-supplementary.pdf]

Supporting information for

Eco-friendly cellulose-supported nickel complex as an efficient and recyclable heterogeneous catalyst for Suzuki cross-coupling reaction

Content

1. **General procedure and Characterization data for products.......................... s3**

2. **Copies of ^{1}H NMR and ^{13}C NMR spectra of the products............................ s10**

3. **Data of GPC s39**

1. General procedure and Characterization data for products

Substituted aryl iodobenzene (0.2 mmol), substituted aryl boronic acid (0.26 mmol), CL-AcPy-Ni (96 mg), and K_3PO_4 (0.6 mmol) were added into 10 mL Shrek tubes, and finally, 1 mL of THF was added, and nitrogen was withdrawn and deflated for three times in double-rowed tubes, and the reaction was carried out at 120 ℃ for 24 h. At the end of the reaction, the solid was separated by centrifugation and washed three times with ethanol, water and anhydrous ethanol to obtain the catalyst, which was dried naturally and reused. The liquid was separated by column chromatography to give the desired products (**3a-3w**).

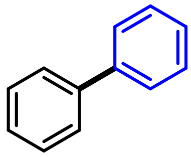

1,1'-biphenyl (**3a**): Following general procedure, (1) the reaction of **1** (Chlorobenzene) (22.5 mg, 0.2 mmol), **2** (Phenylboronic acid) (31.5 mg, 0.26 mmol, 1.3 equiv.); (2) the reaction of **1** (Bromobenzene) (31.4 mg, 0.2 mmol), **2** (Phenylboronic acid) (31.5 mg, 0.26 mmol, 1.3 equiv.); (3) the reaction of **1** (Iodobenzene) (40.8 mg, 0.2 mmol), **2** (Phenylboronic acid) (31.5 mg, 0.26 mmol, 1.3 equiv.). The product was isolated by column chromatography (petrol ether) as white solid (**3a**$_1$, trace; **3a**$_2$, 13.6 mg, 44%; **3a**$_3$, 22.8 mg, 74%). ^{1}H NMR (500 MHz, $CDCl_3$) δ 7.61 (m, 4H), 7.46 (t, *J* = 7.7, 4H), 7.4-7.34 (m, 2H). ^{13}C NMR (126 MHz, $CDCl_3$) δ 141.26, 128.78, 127.28, 127.19.

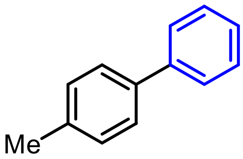

4-methyl-1,1'-biphenyl (**3b**): Following general procedure, the reaction of **1** (4-Iodotoluene) (43.6 mg, 0.2 mmol), **2** (Phenylboronic acid) (31.5 mg, 0.26 mmol, 1.3 equiv.). The product was isolated by column chromatography (petrol ether) as white solid (26.2 mg, 78%). ^{1}H NMR (500 MHz, $CDCl_3$) δ = 7.53 – 7.49 (m, 2H), 7.42 (d, J=8.1, 2H), 7.35 (t, J=7.7, 2H), 7.25 (t, J=7.4, 1H), 7.18 (d, J=6.4, 3H), 2.33 (s, 3H). ^{13}C NMR (125 MHz, $CDCl_3$) δ 141.27, 138.47, 136.79, 129.60, 128.83, 127.28, 127.09, 1226.92, 21.21.

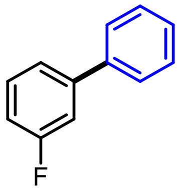

3-fluoro-1,1'-biphenyl (**3c**): Following general procedure, the reaction of **1** (1-Fluoro-3-iodobenzene) (44.4 mg, 0.2 mmol), **2** (Phenylboronic acid) (31.5 mg, 0.26 mmol, 1.3 equiv.). The product was isolated by column chromatography (petrol ether) as white oil (22.7 mg, 66%). ^{1}H NMR (500 MHz, $CDCl_3$) δ 7.58 (m, 2H), 7.40 (m, 5H), 7.29 (m, 1H), 7.04 (m, 1H). ^{13}C NMR (126 MHz, $CDCl_3$) δ 164.18 (d, *J* = 245.5 Hz),

143.56 (d, J = 8.1 Hz), 139.97 (d, J = 1.9 Hz), 130.25 (d, J = 8.4 Hz), 128.89, 127.85, 127.12, 122.79 (d, J = 2.5 Hz), 114.13 (d, J = 21.2 Hz), 113.96 (d, J = 21.2 Hz). ^{19}F NMR (470 MHz, $CDCl_3$) δ = -113.18.

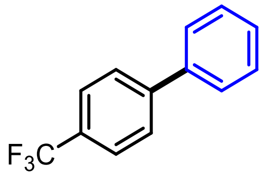

4-(trifluoromethyl)-1,1'-biphenyl (**3d**): Following general procedure, the reaction of **1** (4-Iodobenzotrifluoride) (54 mg, 0.2 mmol), **2** (Phenylboronic acid) (31.6 mg, 0.26 mmol, 1.3 equiv.). The product was isolated by column chromatography (petrol ether) as white oil (35 mg, 79%). ^{1}H NMR (500 MHz, $CDCl_3$) δ 7.70 (s, 4H), 7.61 (m, 2H), 7.48 (t, J = 7.6, 2H), 7.41 (t, J = 7.3, 1H). ^{13}C NMR (126 MHz, $CDCl_3$) δ 143.71, 138.75, 128.45, 128.19, 127.96, 127.15, 126.40, 126.25, 124.72, 124.69, 124.66, 124.63, 124.36, 122.20. ^{19}F NMR (470 MHz, $CDCl_3$) δ = -62.39.

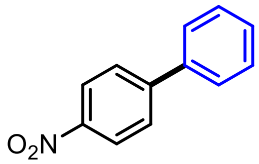

4-nitro-1,1'-biphenyl (**3e**): Following general procedure, the reaction of **1** (1-Iodo-4-nitrobenzene) (50 mg, 0.2 mmol), **2** (Phenylboronic acid) (31.5 mg, 0.26 mmol, 1.3 equiv.). The product was isolated by column chromatography (petrol ether) as white oil (32.2 mg, 81%). ^{1}H NMR (500 MHz, $CDCl_3$) δ 8.29 (d, J = 8.9, 2H), 7.73 (d, J = 8.9, 2H), 7.63 (m, 2H), 7.50 (m, 2H), 7.46 (dd, J = 4.9, 3.6, 1H). ^{13}C NMR (126 MHz, $CDCl_3$) δ = 147.66, 147.10, 138.79, 129.18, 128.94, 127.82, 127.41, 124.13.

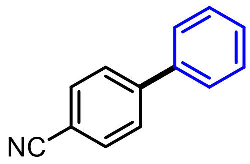

[1,1'-biphenyl]-4-carbonitrile (**3f**): Following general procedure, the reaction of **1** (4-Iodobenzonitrile) (45.8 mg, 0.2 mmol), **2** (Phenylboronic acid) (31.3 mg, 0.26 mmol, 1.3 equiv.). The product was isolated by column chromatography (petrol ether) as white oil (29 mg, 81%). ^{1}H NMR (500 MHz, $CDCl_3$) δ 7.72 (d, J = 8.5, 2H), 7.68 (d, J = 8.5, 2H), 7.59 (m, 2H), 7.48 (m, 2H), 7.41 (dd, J = 8.8, 1.5, 1H). ^{13}C NMR (126 MHz, $CDCl_3$) δ = 145.70, 139.19, 132.62, 129.13, 128.68, 127.75, 127.25, 118.97, 110.92.

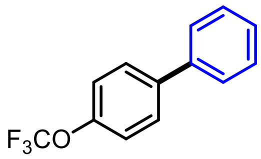

4-(trifluoromethoxy)-1,1'-biphenyl (**3g**): Following general procedure, the reaction of **1** (1-Iado-4-(trifluoromethoxy)benzene) (57.6 mg, 0.2 mmol), **2** (Phenylboronic acid) (31.4 mg, 0.26 mmol, 1.3 equiv.). The product was isolated by column chromatography (petrol ether) as white oil (37.6 mg, 79%). ^{1}H NMR (500 MHz, $CDCl_3$) δ 7.58 (m, 4H), 7.45 (dd, J = 10.9, 4.4, 2H), 7.38 (m, 1H), 7.29 (d, J = 8.0, 2H).^{13}C NMR (126 MHz, $CDCl_3$) δ 148.68, 148.67, 140.00, 139.87, 128.91, 128.48, 127.68, 127.13, 121.57, 121.24, 119.53. ^{19}F NMR (470 MHz, $CDCl_3$) δ = -57.80.

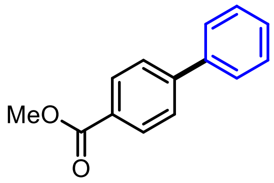

Methyl-[1,1'-biphenyl]-4-carboxylate (**3h**): Following general procedure, the reaction of **1** (Methyl-4-phenylbenzoate) (52 mg, 0.2 mmol), **2** (Phenylboronic acid) (31.5 mg, 0.26 mmol, 1.3 equiv.). The product was isolated by column chromatography (petrol ether) as white oil (35.5 mg, 79%). ^{1}H NMR (500 MHz, $CDCl_3$) δ 8.12 (d, J = 8.3, 2H), 7.68-7.63 (m, 4H), 7.47 (t, J = 7.6, 2H), 7.42 (d, J = 7.6, 1H), 3.95 (s, 3H). ^{13}C NMR (126 MHz, $CDCl_3$) δ 165.98, 144.60, 138.96, 132.77, 129.64, 129.07, 127.89, 127.84, 127.11, 51.11.

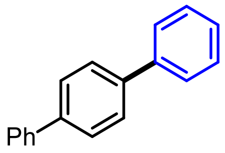

1,1',4',1''-terphenyl (**3i**): Following general procedure, the reaction of **1** (4-Iodobiphenyl) (55 mg, 0.2 mmol), **2** (Phenylboronic acid) (31.5 mg, 0.26 mmol, 1.3 equiv.). The product was isolated by column chromatography (petrol ether) as white oil (13.3 mg, 29%). ^{1}H NMR (500 MHz, $CDCl_3$) δ 7.60 (s, 4H), 7.57 (d, J = 7.3, 4H), 7.38 (t, J = 7.7, 4H), 7.29 (t, J = 7.4, 2H). ^{13}C NMR (126 MHz, $CDCl_3$) δ 140.73, 140.15, 128.84, 127.53, 127.37, 127.08.

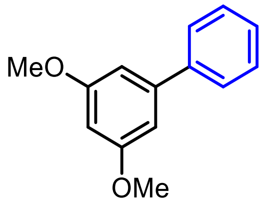

3,5-dimethoxy-1,1'-biphenyl (**3j**): Following general procedure, the reaction of **1** (3-Iodoanisole) (47mg, 0.2 mmol), **2** (Phenylboronic acid) (31.5 mg, 0.26 mmol, 1.3 equiv.). The product was isolated by column chromatography (petrol ether) as white oil (28.3 mg, 77%). ^{1}H NMR (500 MHz, $CDCl_3$) δ 7.58 (m,2H), 7.43 (t, J = 7.6, 2H), 7.35

(t, J = 7.3, 1H), 6.73 (d, J = 2.1, 2H), 6.47 (t, J = 2.1, 1H), 3.85 (s, 6H). ^{13}C NMR (126 MHz, $CDCl_3$) δ 161.05, 143.51, 141.22, 128.72, 127.57, 127.21, 105.48, 99.29, 55.44.

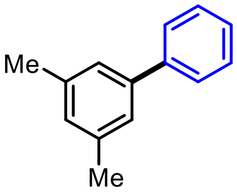

3,5-dimethyl-1,1'-biphenyl (**3k**): Following general procedure, the reaction of **1** (1-Iodo-3,5-dimethylbenzene) (46 mg, 0.2 mmol), **2** (Phenylboronic acid) (31.5 mg, 0.26 mmol, 1.3 equiv.). The product was isolated by column chromatography (petrol ether) as white oil (24.8 mg, 68%). ^{1}H NMR (500 MHz, $CDCl_3$) δ 7.59 (d, J = 7.8, 2H), 7.43 (t, J = 7.6, 2H), 7.33 (t, J = 7.2, 1H), 7.22 (s, 2H), 7.01 (s, 1H), 2.38 (m, 6H). ^{13}C NMR (126 MHz, $CDCl_3$) δ 141.48, 141.28, 138.27, 128.92, 128.66, 127.21, 127.10, 125.13, 21.45.

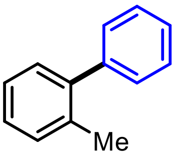

2-methyl-1,1'-biphenyl (**3l**): Following general procedure, the reaction of **1** (2-Iodotoluene) (44.3 mg, 0.2 mmol), **2** (Phenylboronic acid) (31.5 mg, 0.26 mmol, 1.3 equiv.). The product was isolated by column chromatography (petrol ether) as white oil (4.4 mg, 13%). ^{1}H NMR (500 MHz, $CDCl_3$) δ 7.34 (t, J = 7.5, 2H), 7.26 (t, J = 9.0, 3H), 7.17 (m, 5H), 2.20 (s, 3H). ^{13}C NMR (126 MHz, $CDCl_3$) δ 132.31, 130.93, 130.3, 129.79, 129.19, 128.85, 128.06, 127.24, 126.75, 125.75, 20.46.

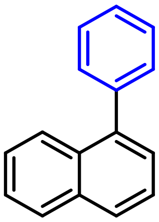

1-phenylnaphthalene (**3m**): Following general procedure, the reaction of **1** (1-Iodonaphthalene) (49 mg, 0.2 mmol), **2** (Phenylboronic acid) (31.5 mg, 0.26 mmol, 1.3 equiv.). The product was isolated by column chromatography (petrol ether) as white oil (16.3 mg, 40%). ^{1}H NMR (500 MHz, $CDCl_3$) δ 7.93 (d, J = 9.4, 2H), 7.88 (d, J = 8.1, 1H), 7.53 (m, 6H), 7.45 (m, 3H). ^{13}C NMR (126 MHz, $CDCl_3$) δ 140.80, 140.30, 133.83, 131.66, 130.11, 128.29, 127.67, 127.27, 126.96, 126.07, 126.05, 125.80, 125.41.

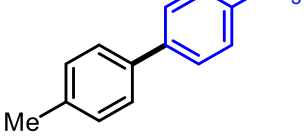

4-methyl-4'-(trifluoromethyl)-1,1'-biphenyl (**3n**): Following general procedure, the reaction of **1** (4-Iodotoluene) (43.6 mg, 0.2 mmol), **2** (4-Trifluoromethylphenylboronic acid) (67 mg, 0.26 mmol, 1.3 equiv.). The product was

isolated by column chromatography (petrol ether) as a colorless solid (33 mg, 70%). ^{1}H NMR (500 MHz, $CDCl_3$) δ 7.69 (s, 4H), 7.51 (d, *J* = 8.1, 2H), 7.29 (d, *J* = 7.9, 2H), 2.43 (s, 3H). ^{13}C NMR (126 MHz, $CDCl_3$) δ 144.66, 138.17, 136.88, 129.72, 129.16, 128.90, 128.62, 127.19, 127.12, 125.72, 125.69, 125.66, 125.63, 21.17. ^{19}F NMR (470 MHz, $CDCl_3$) δ = -62.34.

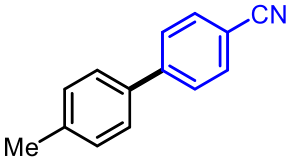

4'-methyl-[1,1'-biphenyl]-4-carbonitrile (**3o**): Following general procedure, the reaction of **1** (4-Iodotoluene) (43.4 mg, 0.2 mmol), **2** (4-Cyanophenylboronic acid) (56.2 mg, 0.26 mmol, 1.3 equiv.). The product was isolated by column chromatography (petrol ether/ethyl acetate = 20:1) as white solid (31 mg, 81%). ^{1}H NMR (500 MHz, $CDCl_3$) δ 7.71 (d, *J* = 8.3, 2H), 7.67 (d, *J* = 8.3, 2H), 7.49 (d, *J* = 8.1, 2H), 7.29 (d, *J* = 8.0, 2H), 2.42 (s, 3H). ^{13}C NMR (126 MHz, $CDCl_3$) δ 144.57, 137.72, 135.24, 131.54, 128.81, 126.43, 126.03, 118.02, 109.50, 20.16.

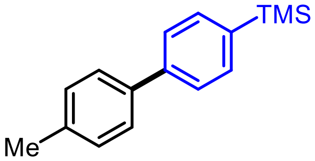

trimethyl(4'-methyl-[1,1'-biphenyl]-4-yl)silane (**3p**): Following general procedure, the reaction of **1** (4-Iodotoluene) (43.5 mg, 0.2 mmol), **2** (4-Triemthylsilylphenylboronic acid) (68.3 mg, 0.26 mmol, 1.3 equiv.). The product was isolated by column chromatography (petrol ether) as colorless oil (29 mg, 61%). ^{1}H NMR (500 MHz, $CDCl_3$) δ 7.51 (m, 4H), 7.43 (m, 2H), 7.18 (s, 1H), 7.17 (s, 1H), 2.32 (s, 3H), 0.22 (d, *J* = 2.9 Hz, 9H). ^{13}C NMR (126 MHz, $CDCl_3$) δ 142.60, 139.92, 139.34, 138.19, 134.86, 130.55, 128.07, 127.38, 22.19, 0.00.

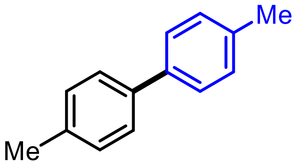

4,4'-dimethyl-1,1'-biphenyl (**3q**): Following general procedure, the reaction of **1** (4-Iodotoluene) (43.6 mg, 0.2 mmol), **2** (4-Tolylboronic acid) (53 mg, 0.26 mmol, 1.3 equiv.). The product was isolated by column chromatography (petrol ether) as White solid (20.4 mg, 56%). ^{1}H NMR (500 MHz, $CDCl_3$) δ 7.40 (d, *J* = 8.1 Hz, 4H), 7.16 (d, *J* = 8.0 Hz, 4H), 2.31 (s, 6H). ^{13}C NMR (126 MHz, $CDCl_3$) δ 138.31, 136.73, 129.46, 126.84, 21.12.

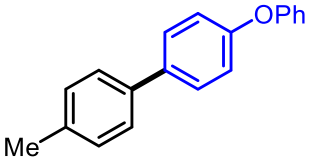

4-methyl-4'-phenoxy-1,1'-biphenyl (**3r**): Following general procedure, the

reaction of **1** (4-Iodotoluene) (43.6 mg, 0.2 mmol), **2** (4-Phenoxybenzeneboronic acid) (72.2 mg, 0.26 mmol, 1.3 equiv.). The product was isolated by column chromatography (petrol ether) as White solid (13.5 mg, 26%). ^{1}H NMR (500 MHz, $CDCl_3$) δ 7.46 (m, 2H), 7.39 (d, *J* = 8.1 Hz, 2H), 7.28 (m, 2H), 7.16 (d, *J* = 7.4 Hz, 2H), 7.04 (t, *J* = 7.4 Hz, 1H), 6.98 (m, 4H), 2.31 (s, 3H). ^{13}C NMR (126 MHz, $CDCl_3$) δ 156.18, 155.51, 136.63, 135.75, 135.22, 128.73, 128.46, 127.18, 125.70, 122.25, 118.02, 117.90, 20.05.

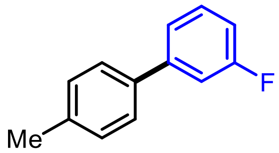

3-fluoro-4'-methyl-1,1'-biphenyl (**3s**): Following general procedure, the reaction of **1** (4-Iodotoluene) (43.2 mg, 0.2 mmol), **2** (3-Fluorophenylboronic acid) (54 mg, 0.26 mmol, 1.3 equiv.). The product was isolated by column chromatography (petrol ether) as white oil (7.3 mg, 20%). ^{1}H NMR (500 MHz, $CDCl_3$) δ 7.40 (d, *J* = 8.0, 2H), 7.29 (m, 2H), 7.18 (m, 3H), 6.93 (dd, *J* = 10.7, 5.5, 1H), 2.33 (s, 3H). ^{13}C NMR (126 MHz, $CDCl_3$) δ 164.19 (d, *J* = 243.8 Hz), 143.48 (d, *J* = 7.6 Hz), 137.74, 137.08 (d, *J* = 2.5 Hz), 130.18 (d, *J* = 8.4 Hz), 129.61, 126.93, 122.56 (d, *J* = 2.9 Hz), 113.87 (d, *J* = 21.5 Hz), 113.70 (d, *J* = 21.0 Hz), 21.13. ^{19}F NMR (470 MHz, $CDCl_3$) δ = -113.29.

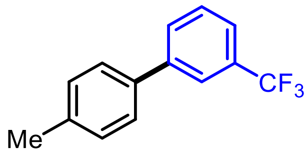

4'-methyl-3-(trifluoromethyl)-1,1'-biphenyl (**3t**): Following general procedure, the reaction of **1** (4-Iodotoluene) (43.2 mg, 0.2 mmol), **2** (3-(Trifluoromethyl)phenylboronic acid) (67.7 mg, 0.26 mmol, 1.3 equiv.). The product was isolated by column chromatography (petrol ether) as white oil (18.9 mg, 72%). ^{1}H NMR (500 MHz, $CDCl_3$) δ 7.82 (s, 1H), 7.75 (d, *J* = 7.5 Hz, 1H), 7.59-7.49 (m, 2H), 7.50 (d, *J* = 8.1 Hz, 2H), 7.28 (d, *J* = 7.9 Hz, 2H), 2.41 (s, 3H). ^{13}C NMR (126 MHz, $CDCl_3$) δ 141.93, 137.97, 136.88, 130.22, 129.73, 129.19, 127.02, 123.77, 123.74, 123.71, 123.68, 123.66, 123.63, 123.60, 21.15. ^{19}F NMR (470 MHz, $CDCl_3$) δ = -62.59.

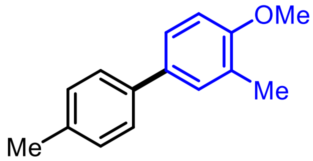

4-methoxy-3,4'-dimethyl-1,1'-biphenyl (**3u**): Following general procedure, the reaction of **1** (4-Iodotoluene) (43.4 mg, 0.2 mmol), **2** (4-Methoxy-3-Methylphenylboronic acid) (60.7 mg, 0.26 mmol, 1.3 equiv.). The product was isolated by column chromatography (petrol ether) as white solid (12.5 mg, 40%). ^{1}H NMR (500 MHz, $CDCl_3$) δ 7.46 (m, 2H), 7.39 (m, 2H), 7.23 (d, *J* = 8.1, 2H), 6.89 (m, 1H), 3.87 (s, 3H), 2.39 (s, 3H), 2.29 (s, 3H). ^{13}C NMR (126 MHz, $CDCl_3$) δ 157.19, 138.21, 136.21, 133.34, 129.40, 129.35, 126.85, 126.62, 125.18, 110.18, 55.45, 21.07, 16.42.

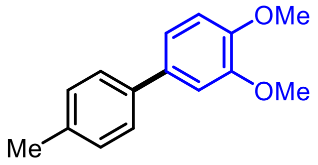

3,4-dimethoxy-4'-methyl-1,1'-biphenyl (**3v**): Following general procedure, the reaction of **1** (4-Iodotoluene) (43.2 mg, 0.2 mmol), **2** (3,4-Dimethoxyphenylboronic acid) (65 mg, 0.26 mmol, 1.3 equiv.). The product was isolated by column chromatography (petrol ether) as white oil (22.2 mg, 39%). ^{1}H NMR (500 MHz, $CDCl_3$) δ 7.45 (d, *J* = 8.1, 2H), 7.23 (d, *J* = 8.0, 2H), 7.13 (dd, *J* = 8.2, 2.1, 1H), 7.09 (d, *J* = 2.0, 1H), 6.93 (d, *J* = 8.2, 1H), 3.94 (s, 3H), 3.92 (s, 3H), 2.39 (s, 3H). ^{13}C NMR (126 MHz, $CDCl_3$) δ 149.13, 148.41, 138.21, 136.60, 134.26, 129.47, 126.73, 119.19, 111.50, 110.36, 56.00, 55.94, 21.09.

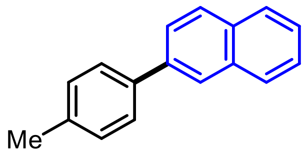

2-(*p*-tolyl)naphthalene (**3w**): Following general procedure, the reaction of **1** (4-Iodotoluene) (43.5 mg, 0.2 mmol), **2** (2-Naphthaleneboronic acid) (62 mg, 0.26 mmol, 1.3 equiv.), The product was isolated by column chromatography (petrol ether) as white oily (24 mg, 56%). ^{1}H NMR (500 MHz, $CDCl_3$) δ 7.95 (s, 1H), 7.80 (m, 3H), 7.67 (dd, J = 8.5, 1.7 Hz, 1H), 7.55 (d, *J* = 8.1 Hz, 2H), 7.41 (m, 2H), 7.22 (d, *J* = 7.9 Hz, 2H), 2.35 (s, 3H). ^{13}C NMR (126 MHz, $CDCl_3$) δ 138.49, 138.24, 137.18, 133.73, 132.51, 129.61, 128.36, 128.15, 127.64, 127.27, 126.24, 125.78, 125.57, 125.44, 22.28.

2. Copies of ^{1}H NMR and ^{13}C NMR spectra of the products

^{1}H NMR of 1,1'-biphenyl (3a)

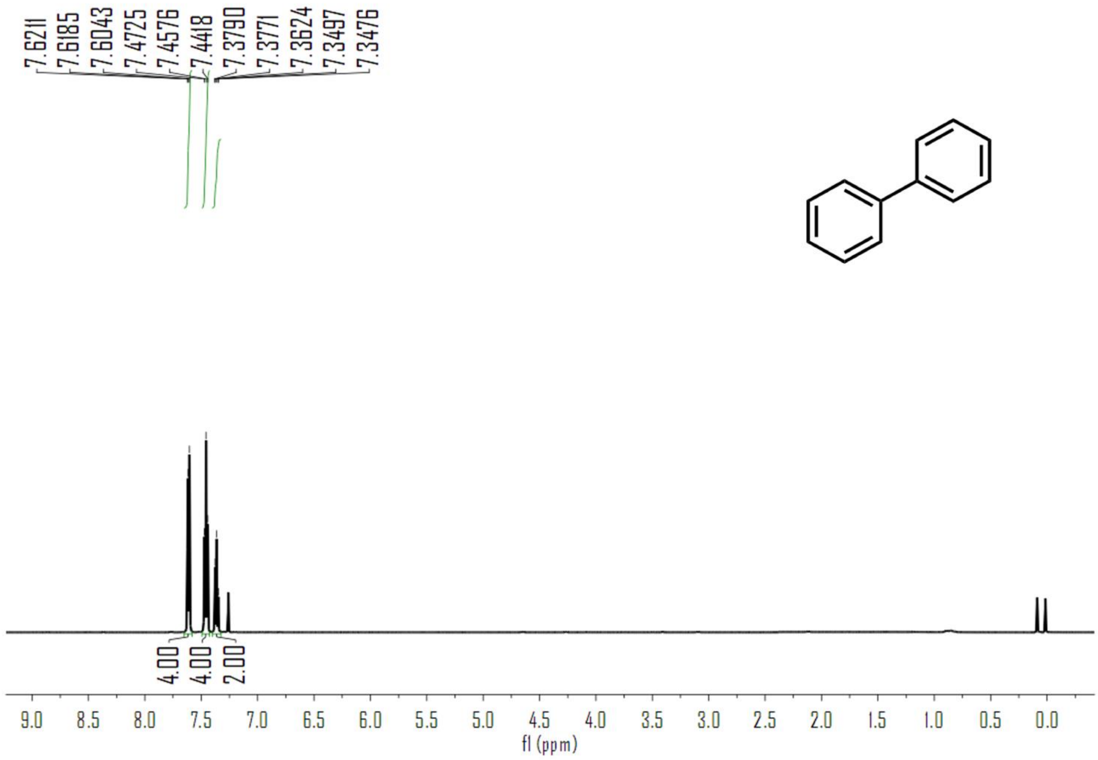

^{13}C NMR of 1,1'-biphenyl (3a)

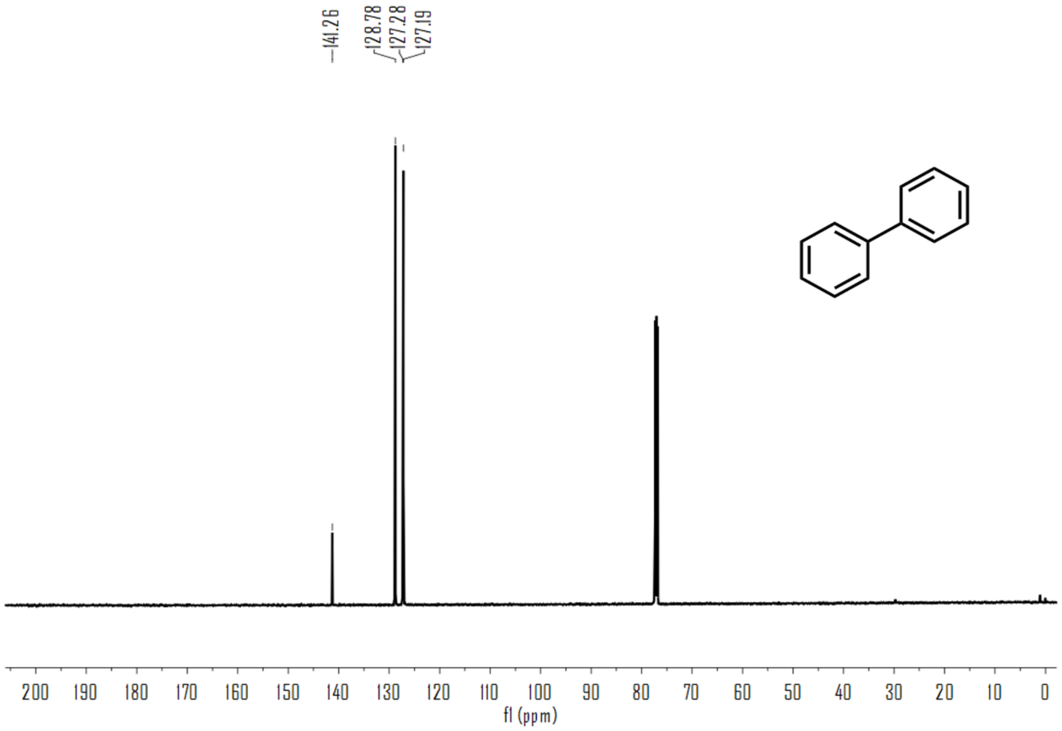

^{1}H NMR of 4-methyl-1,1'-biphenyl (3b)

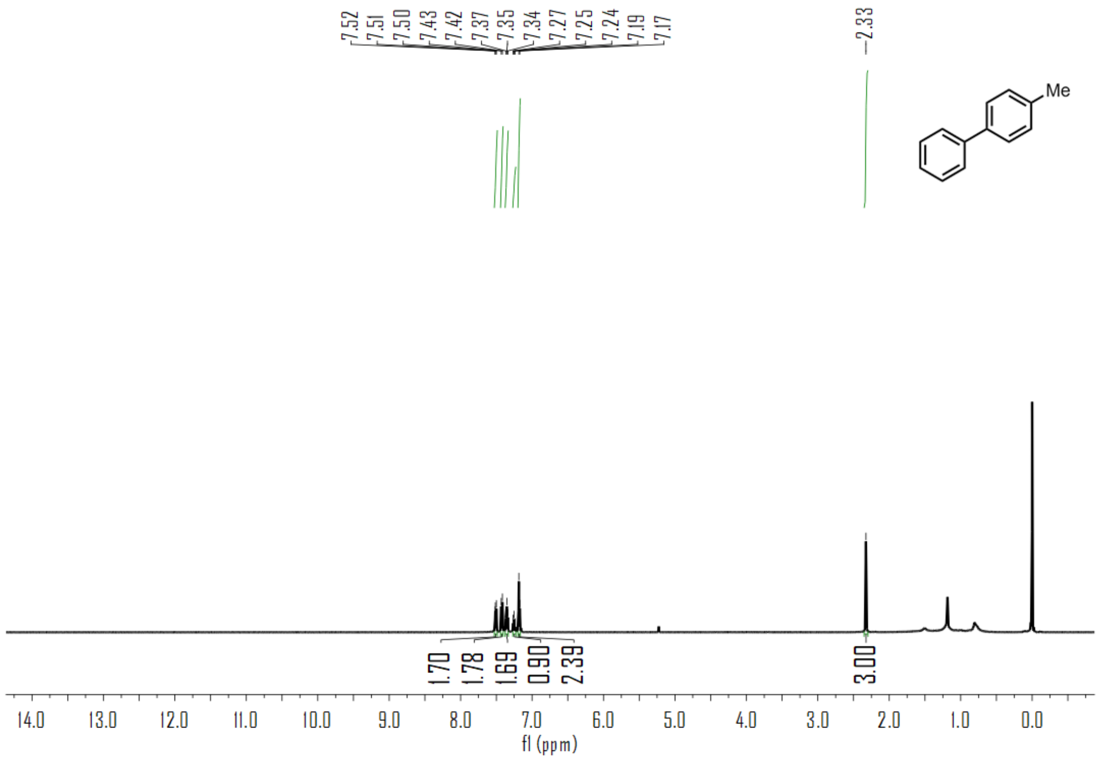

^{13}C NMR of 4-methyl-1,1'-biphenyl (3b)

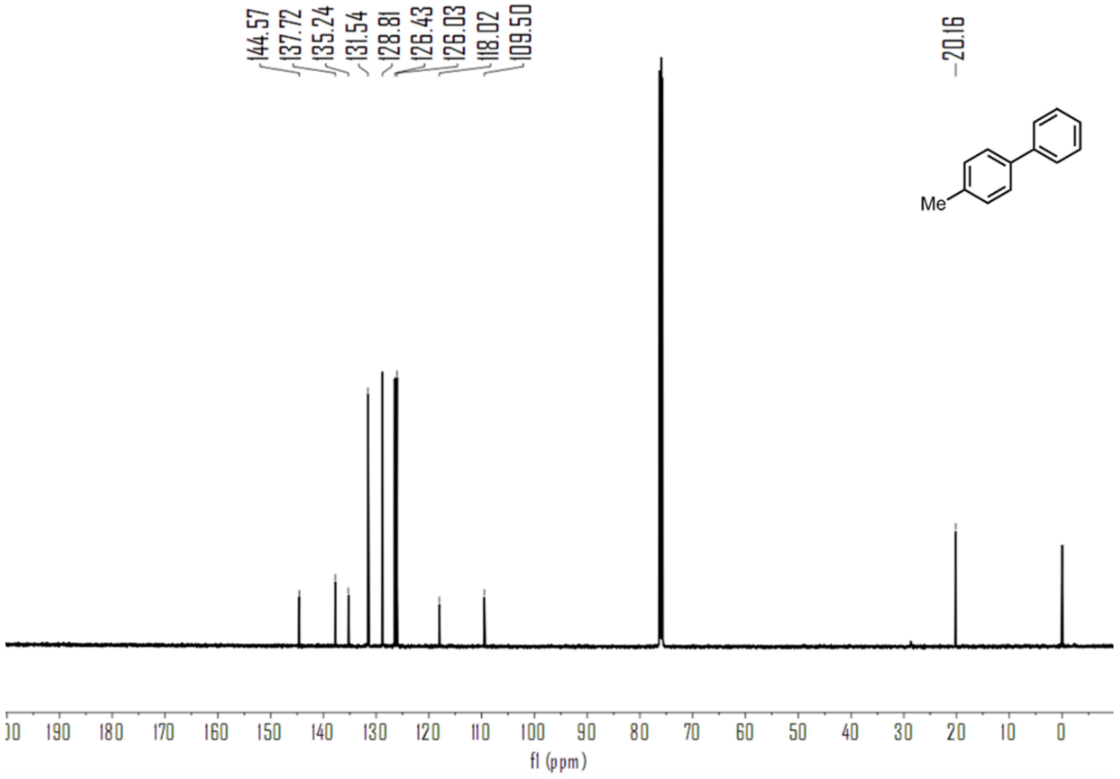

^{1}H NMR of 3-fluoro-1,1'-biphenyl (3c)

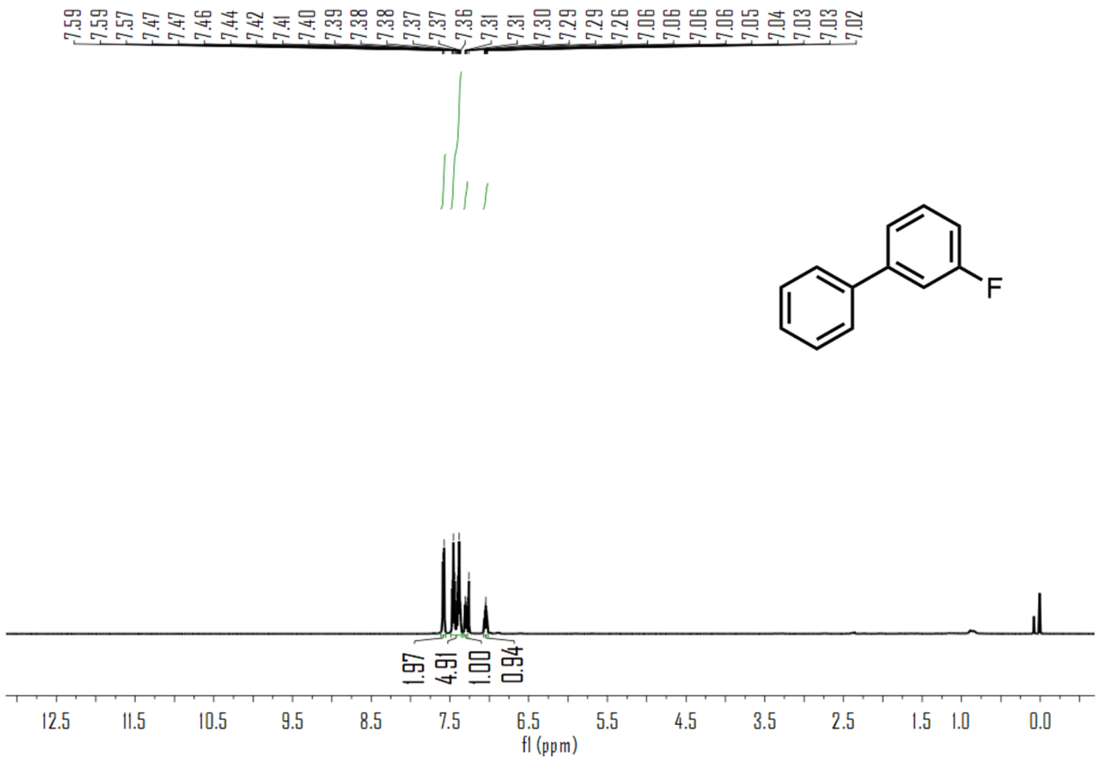

^{13}C NMR of 3-fluoro-1,1'-biphenyl (3c)

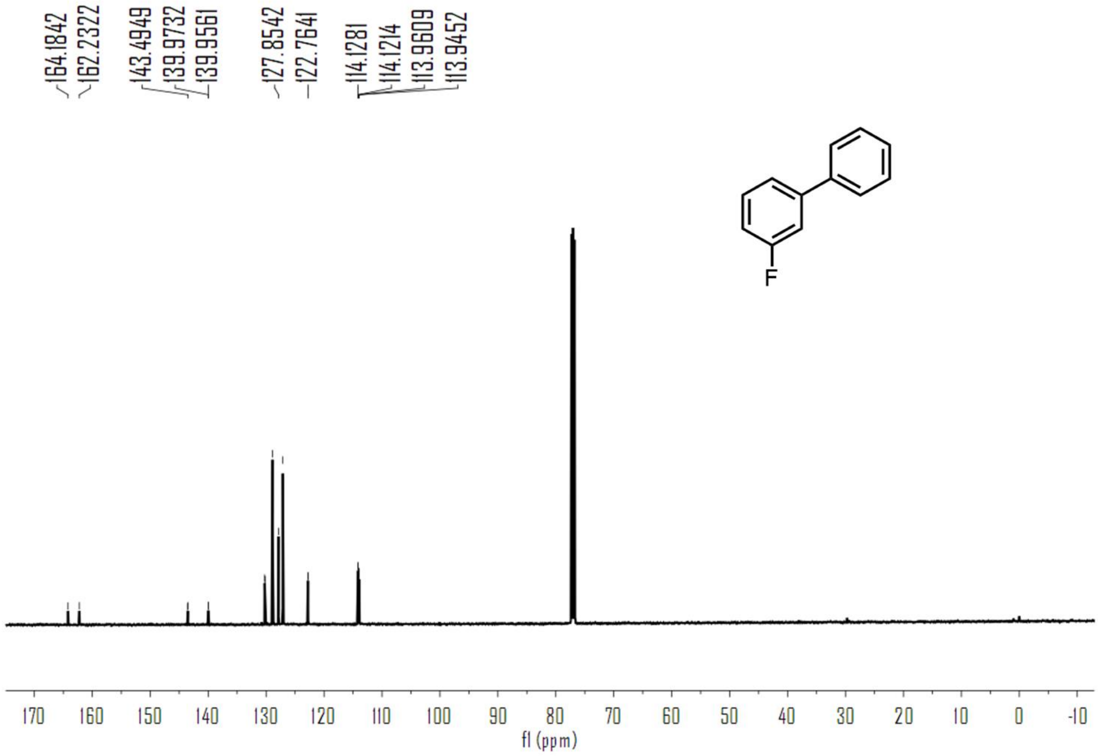

^{19}F NMR of 3-fluoro-1,1'-biphenyl (3c)

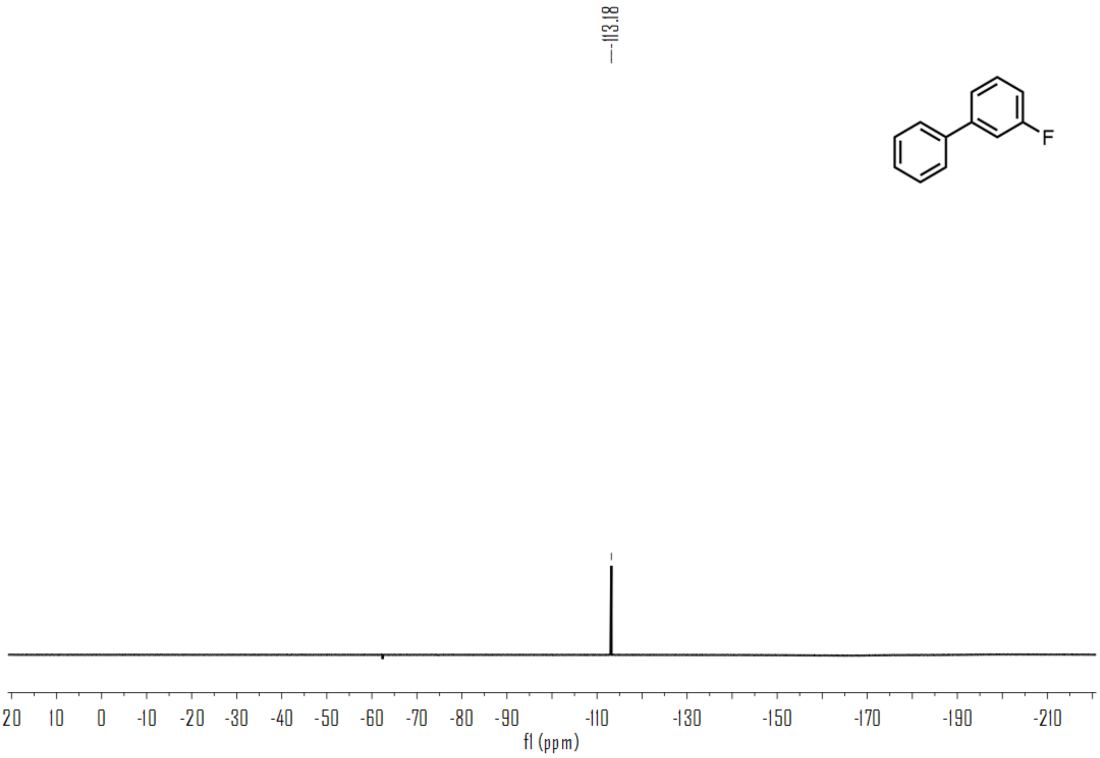

^{1}H NMR of 4-(trifluoromethyl)-1,1'-biphenyl (3d)

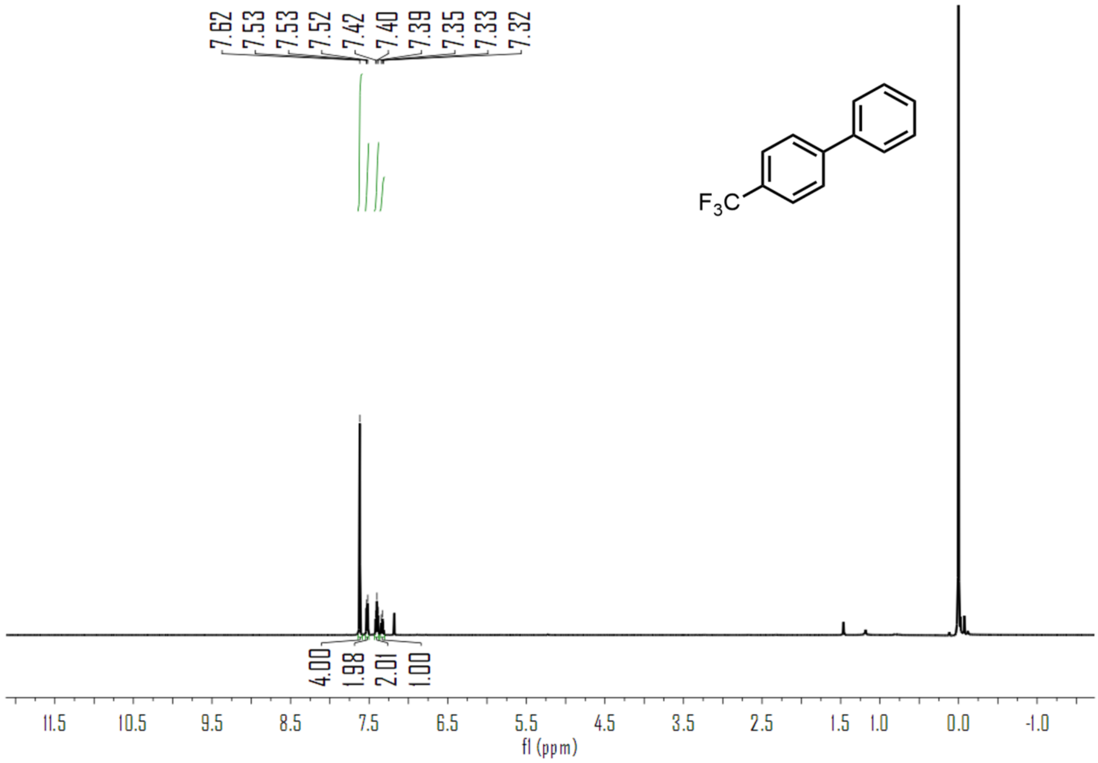

^{13}C NMR of 4-(trifluoromethyl)-1,1'-biphenyl (3d)

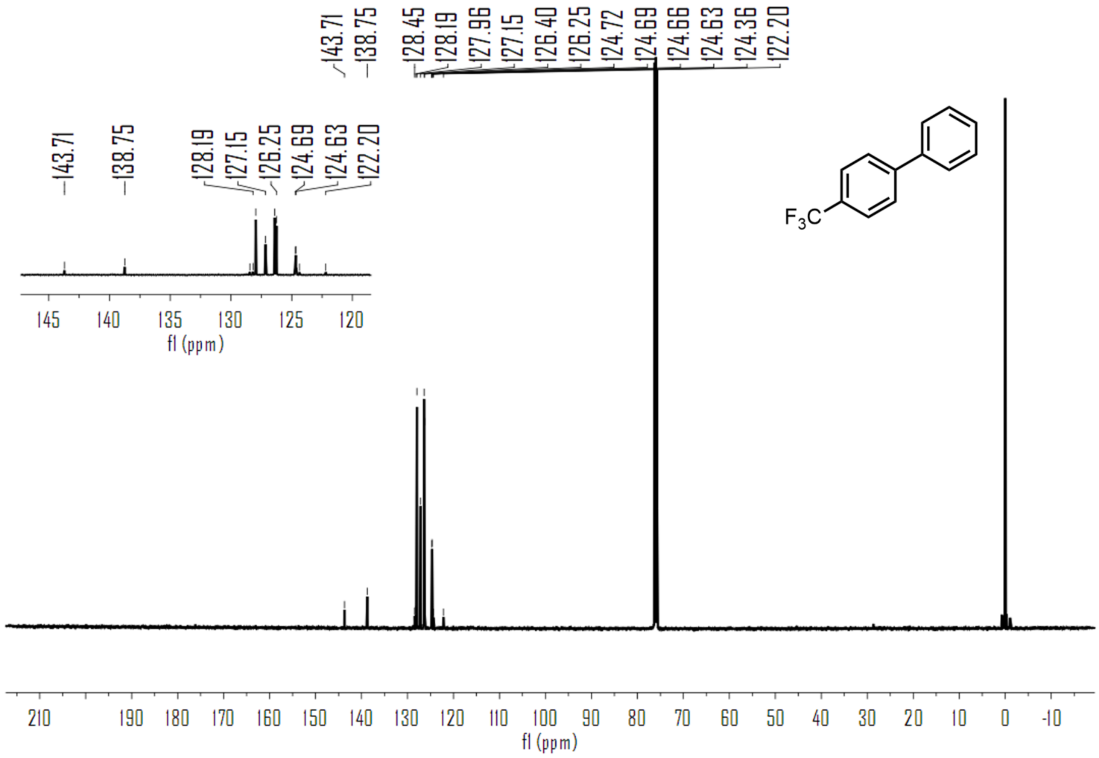

^{19}F NMR of 4-(trifluoromethyl)-1,1'-biphenyl (3d)

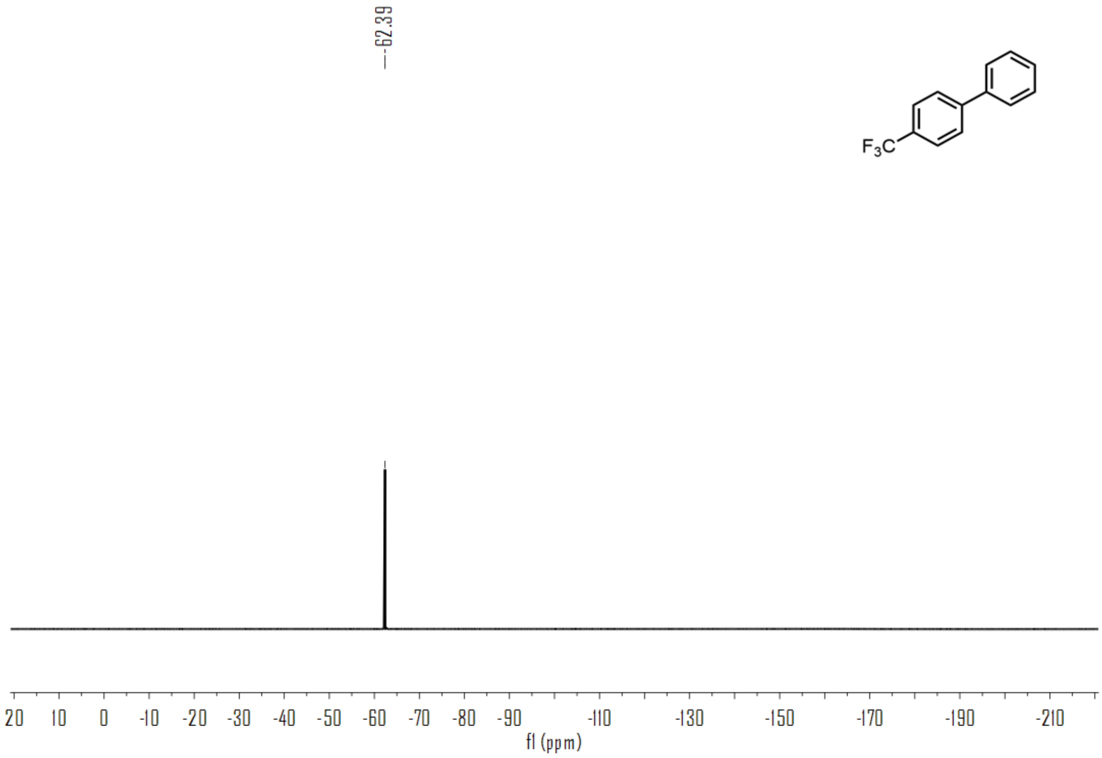
-62.39
F3C
20
10
0
-10
-20
-30
-40
-50
-60
-70
-80
-90
-110
-130
-150
-170
-190
-210
f1 (ppm)

^{1}H NMR of 4-nitro-1,1'-biphenyl (3e)

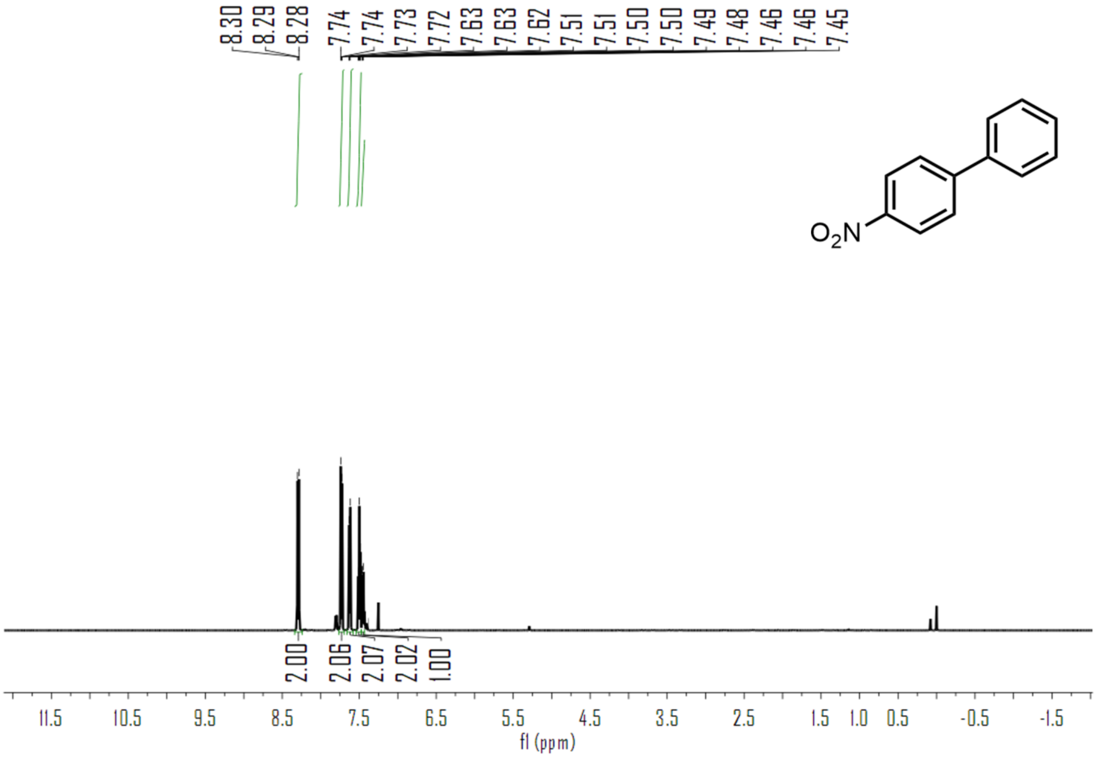

^{13}C NMR of 4-nitro-1,1'-biphenyl (3e)

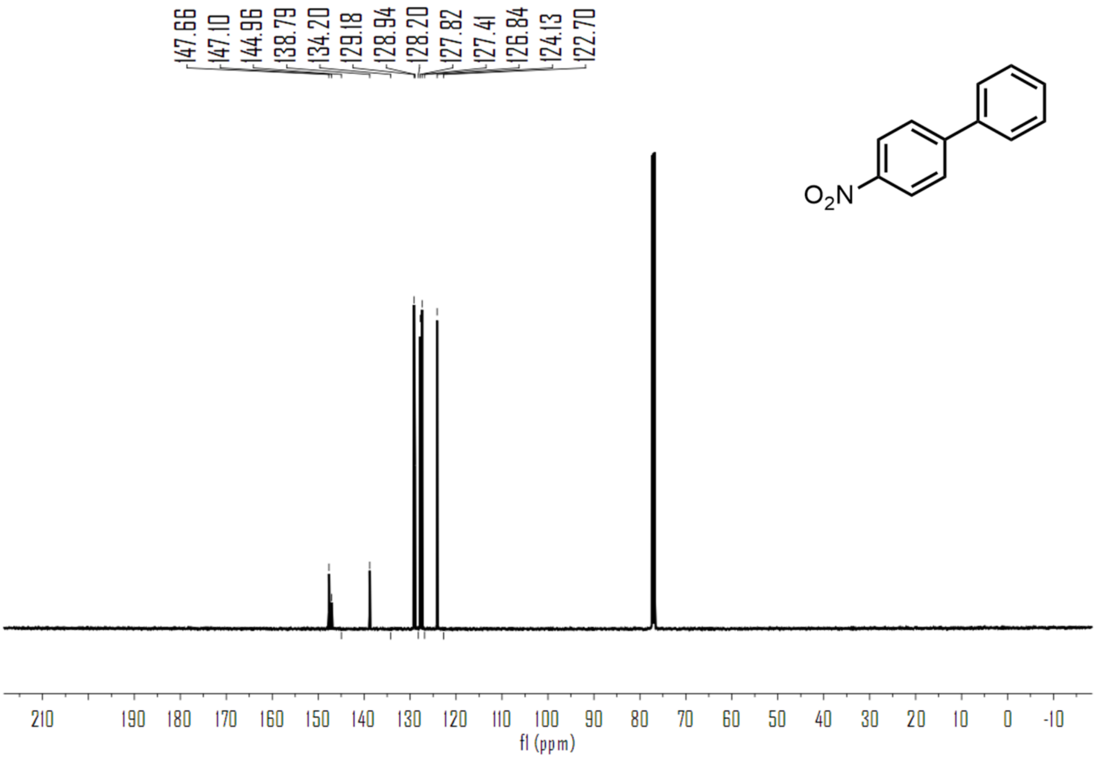

^{1}H NMR of [1,1'-biphenyl]-4-carbonitrile (3f)

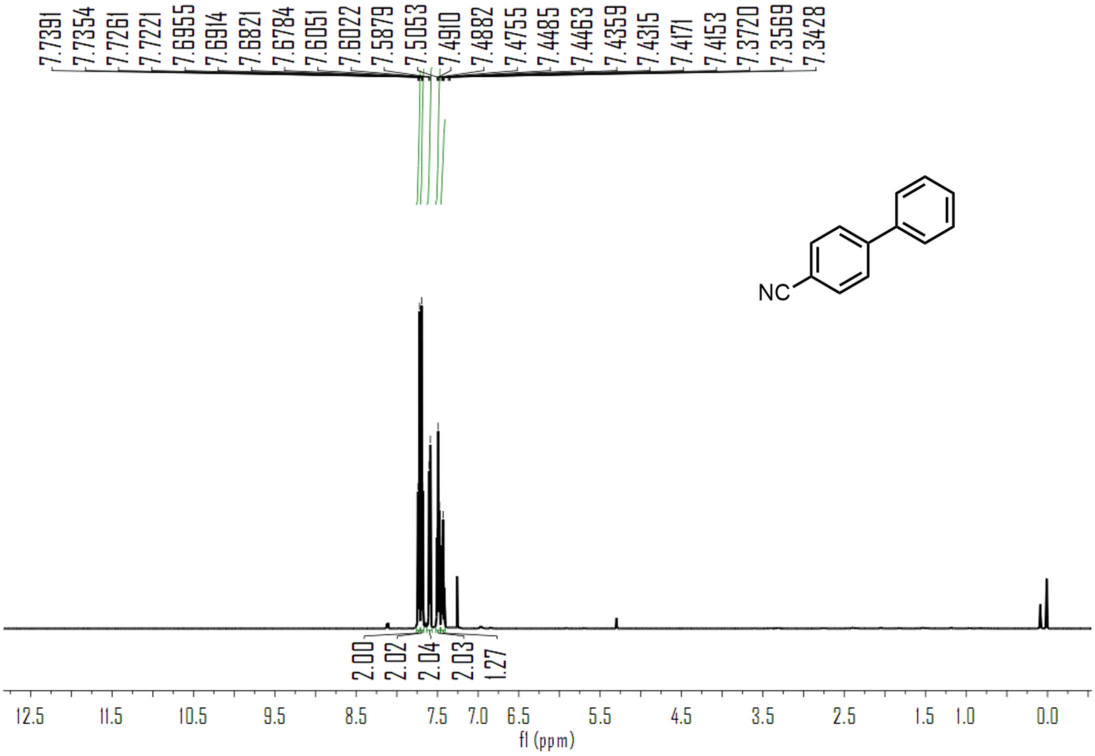

^{13}C NMR of [1,1'-biphenyl]-4-carbonitrile (3f)

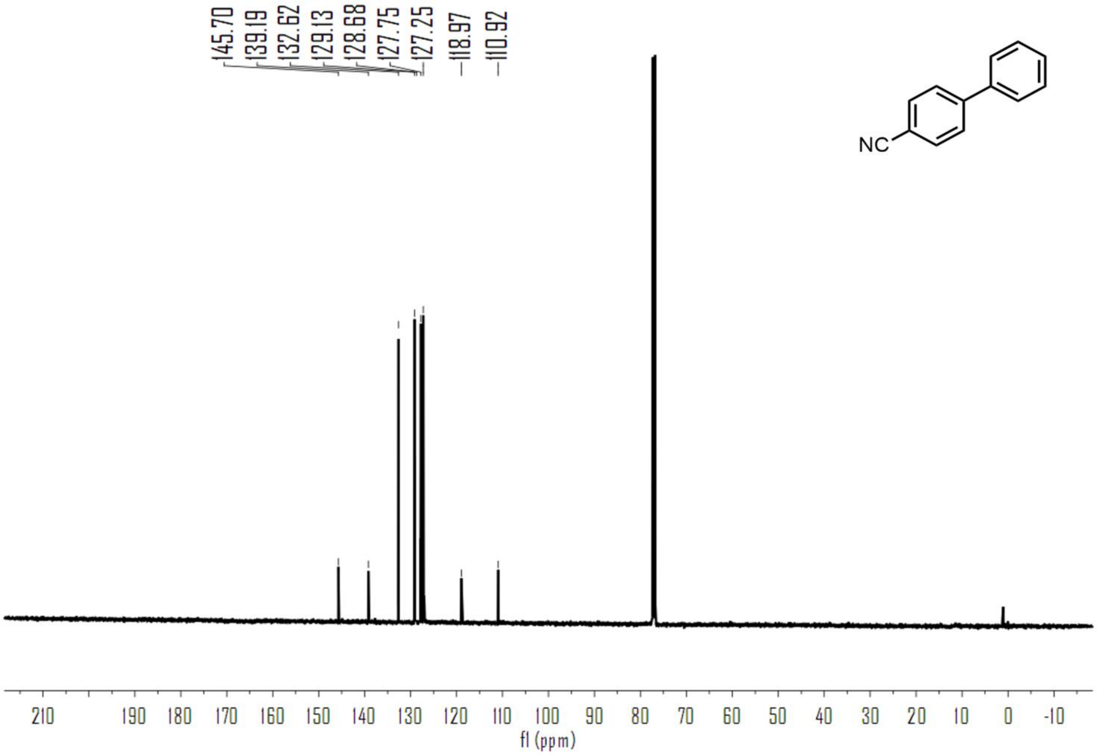

^{1}H NMR of 4-nitro-1,1'-biphenyl (3g)

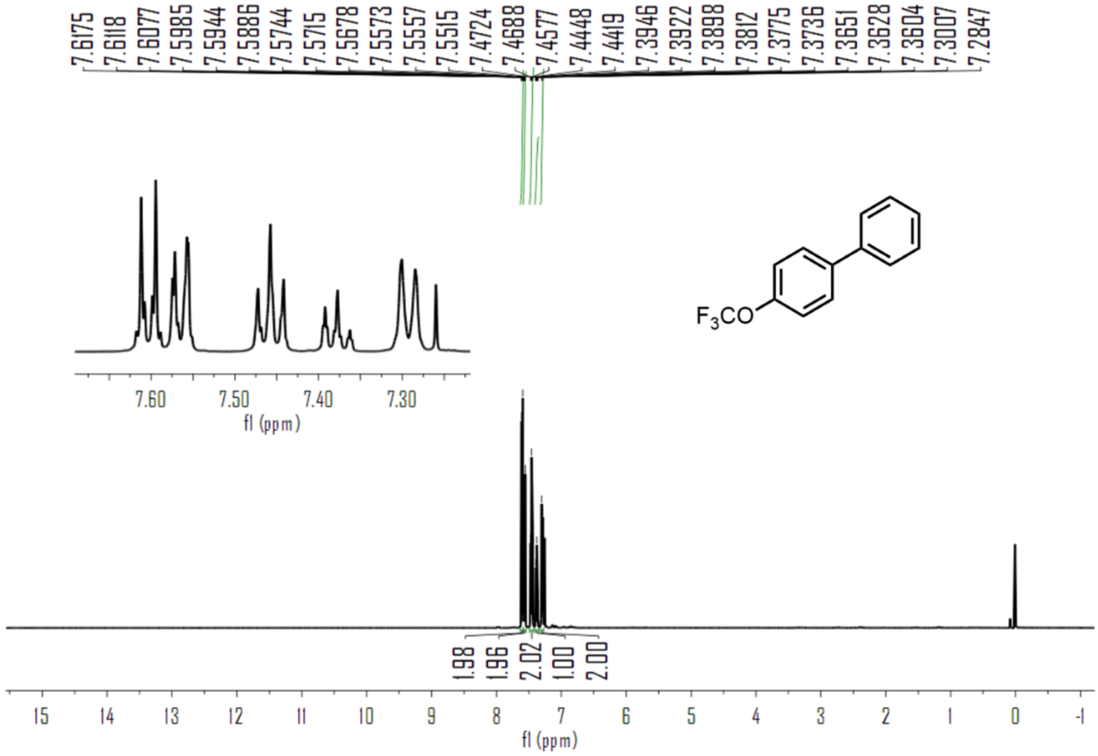

^{13}C NMR of 4-nitro-1,1'-biphenyl (3g)

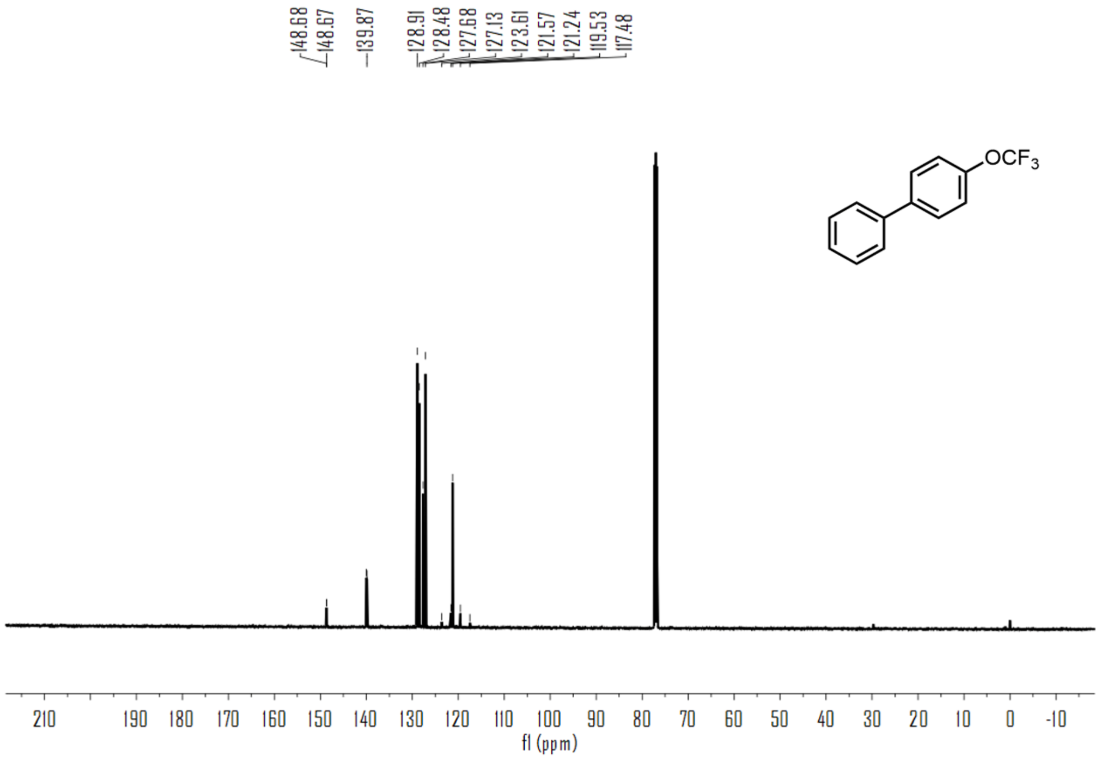

^{19}F NMR of 4-nitro-1,1'-biphenyl (3g)

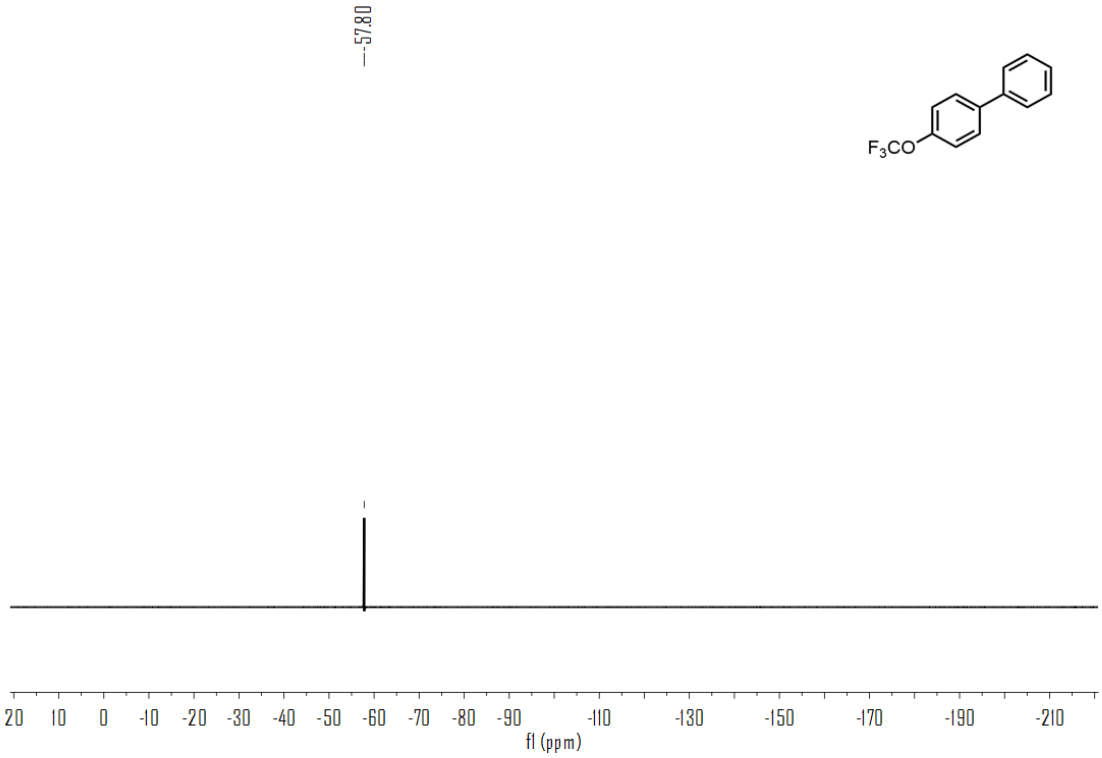

-57.80
F3CO
20
10
0
-10
-20
-30
-40
-50
-60
-70
-80
-90
-110
-130
-150
-170
-190
-210
f1 (ppm)

^{1}H NMR of methyl [1,1'-biphenyl]-4-carboxylate (3h)

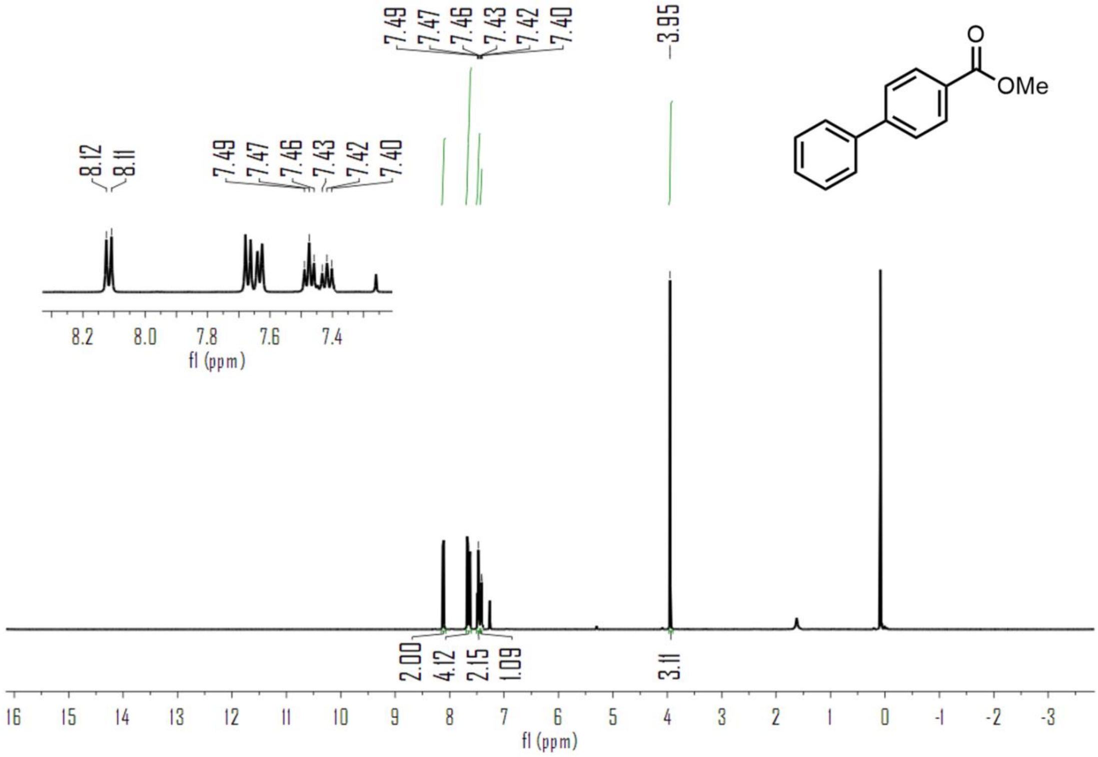

^{13}C NMR of methyl [1,1'-biphenyl]-4-carboxylate (3h)

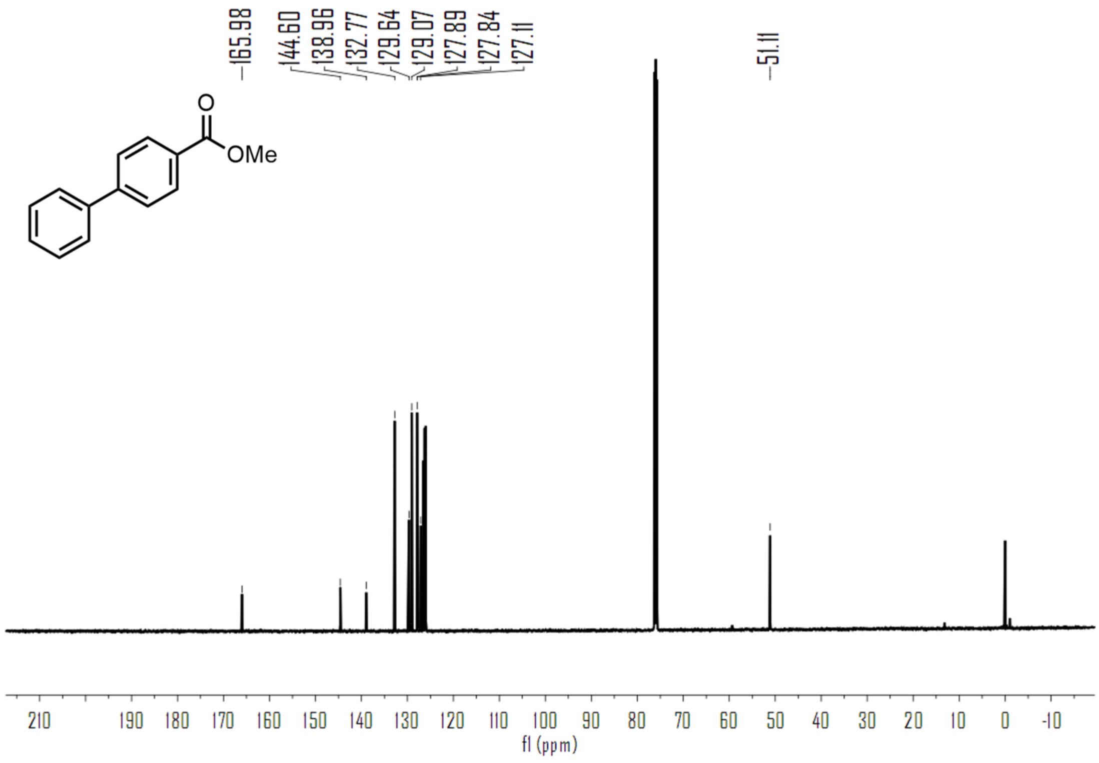

^{1}H NMR of 1,1',4',1''-terphenyl (3i)

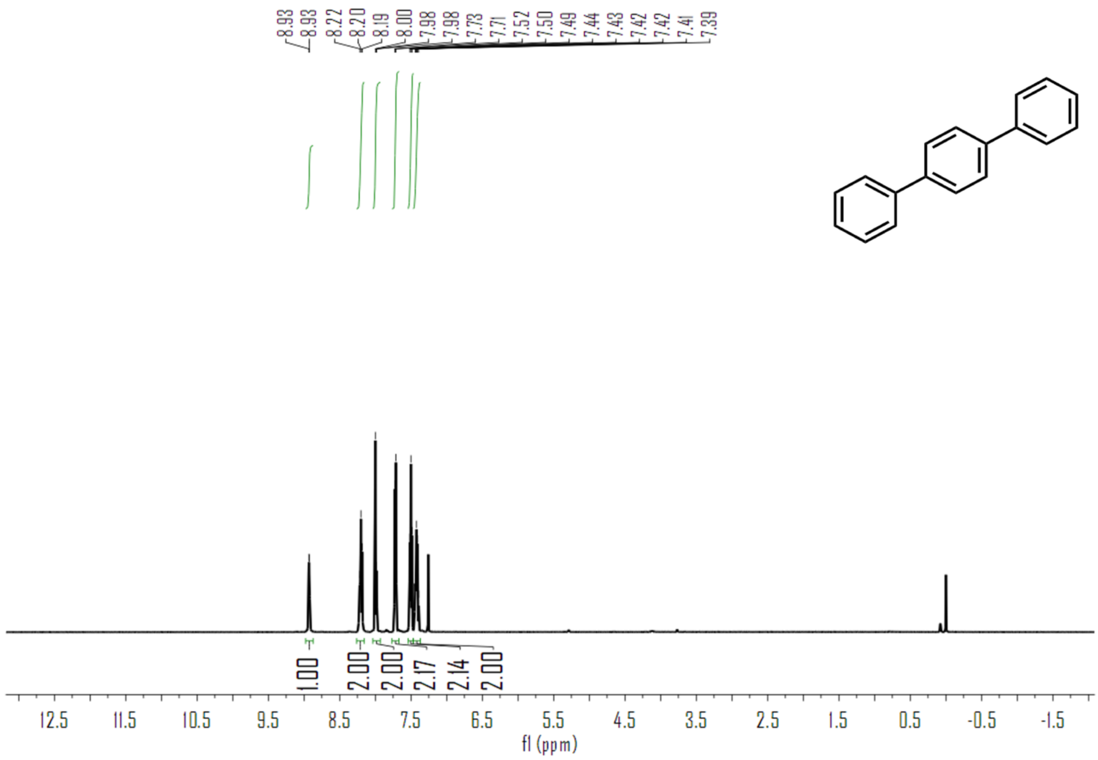

^{13}C NMR of 1,1',4',1''-terphenyl (3i)

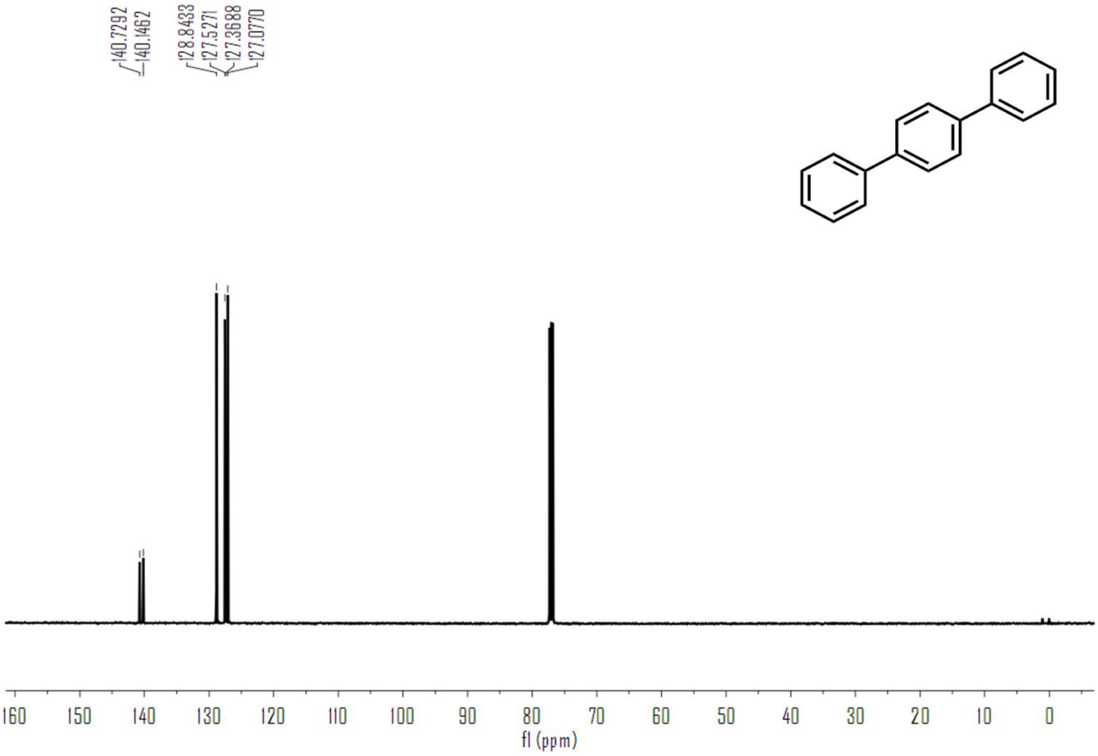

^{1}H NMR of 3-methoxy-1,1'-biphenyl (3j)

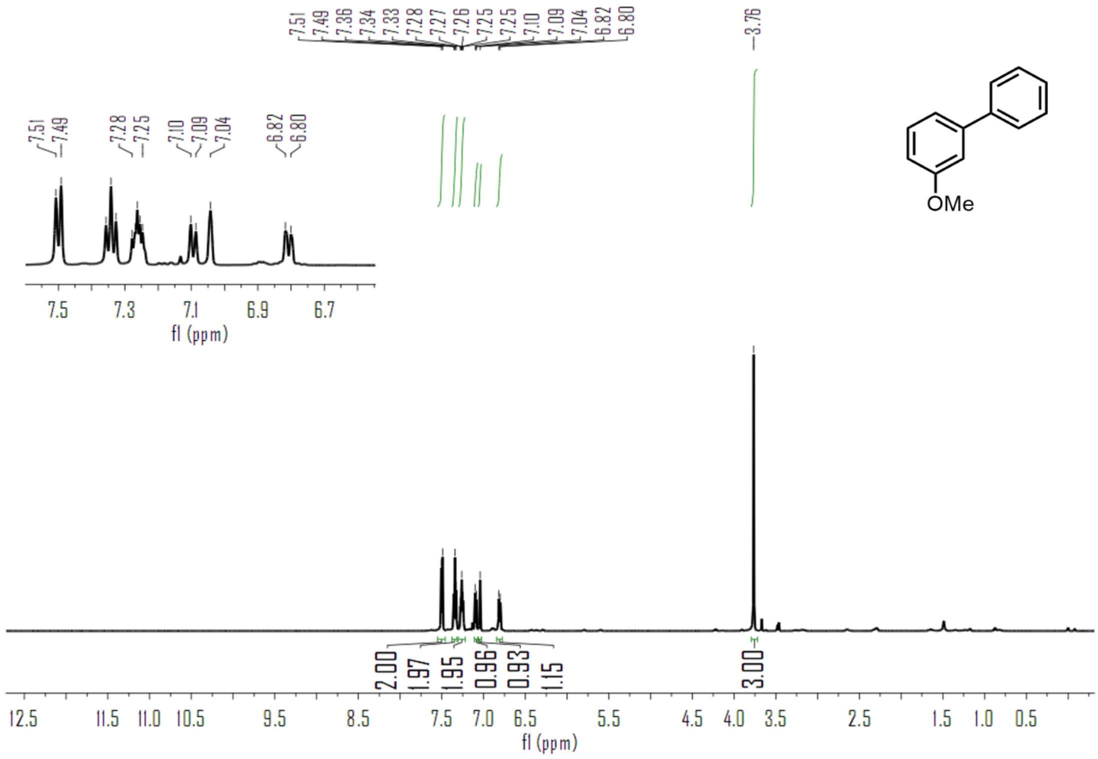

^{13}C NMR of 3-methoxy-1,1'-biphenyl (3j)

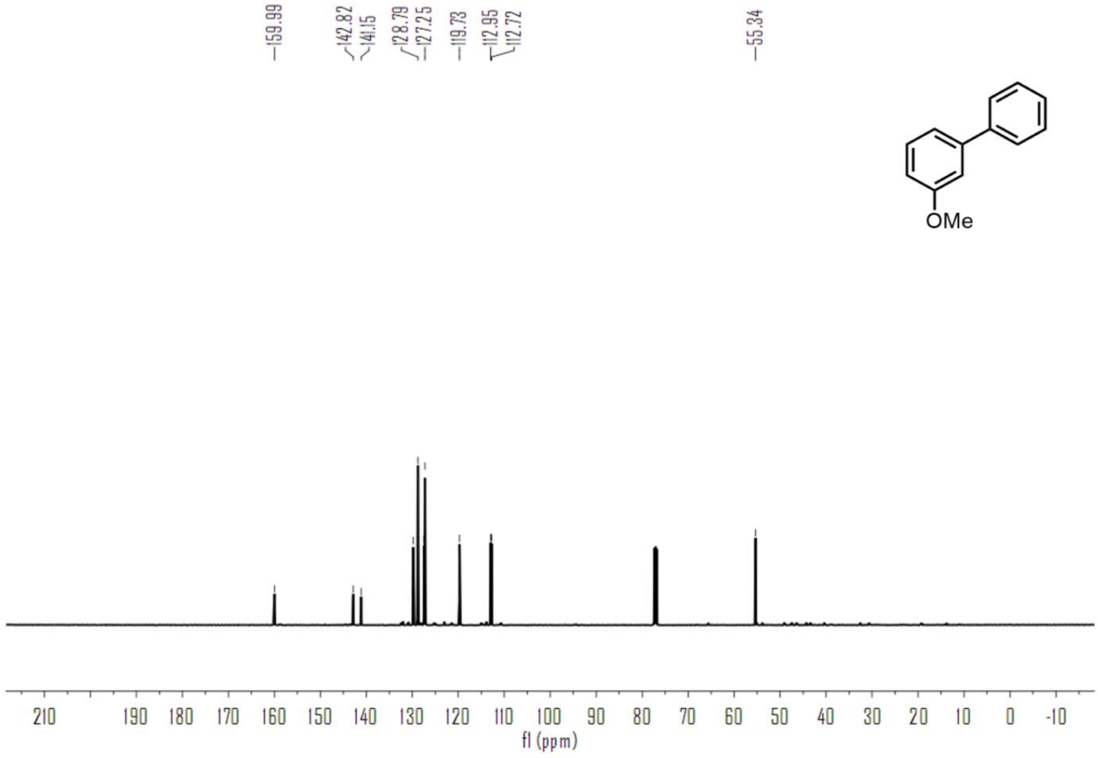

^{1}H NMR of 3,5-dimethyl-1,1'-biphenyl (3k)

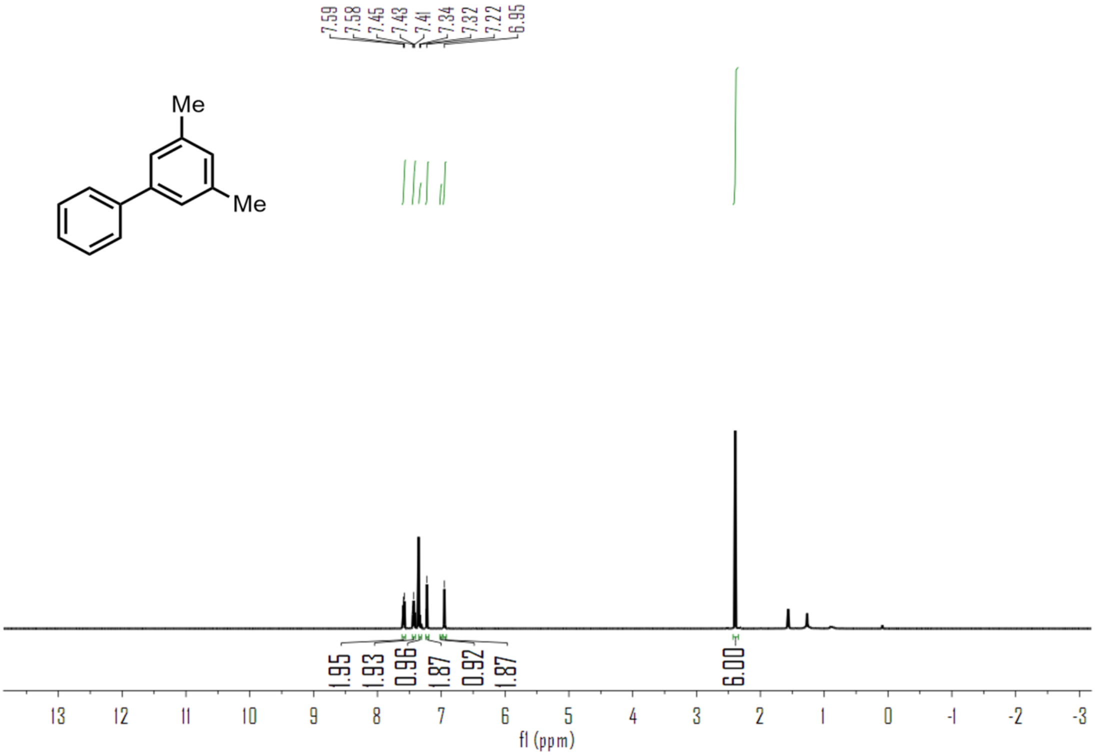

^{13}C NMR of 3,5-dimethyl-1,1'-biphenyl (3k)

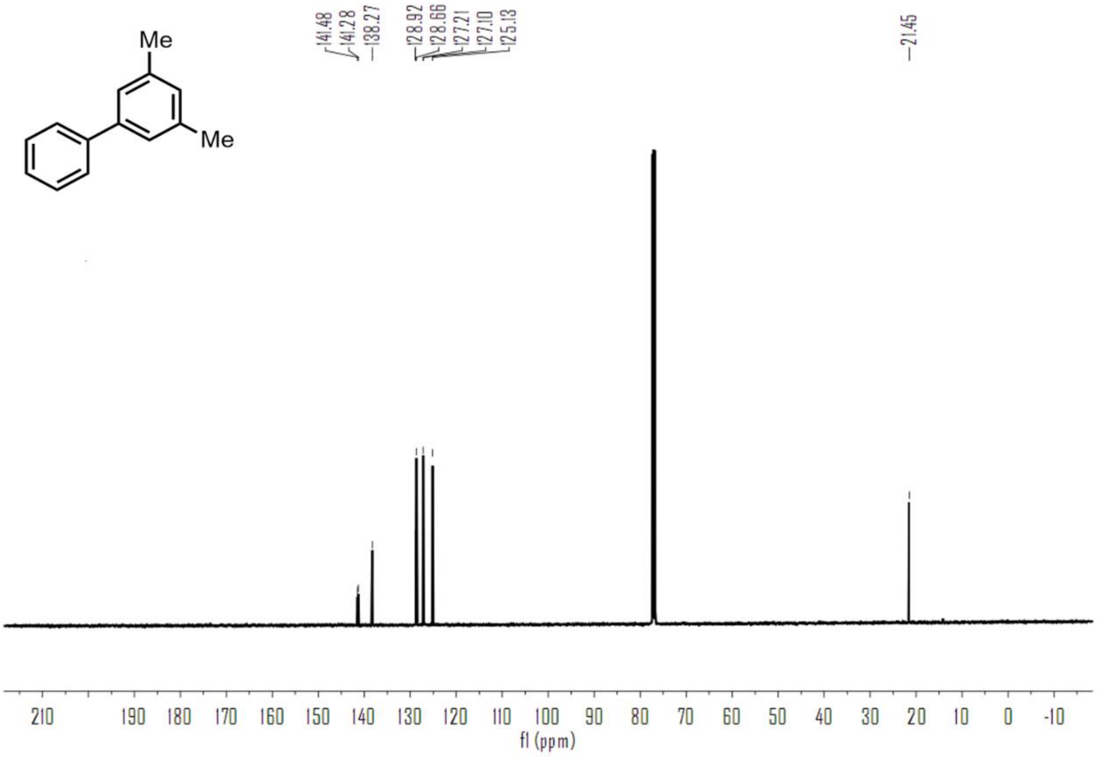

^{1}H NMR of 2-methyl-1,1'-biphenyl (3l)

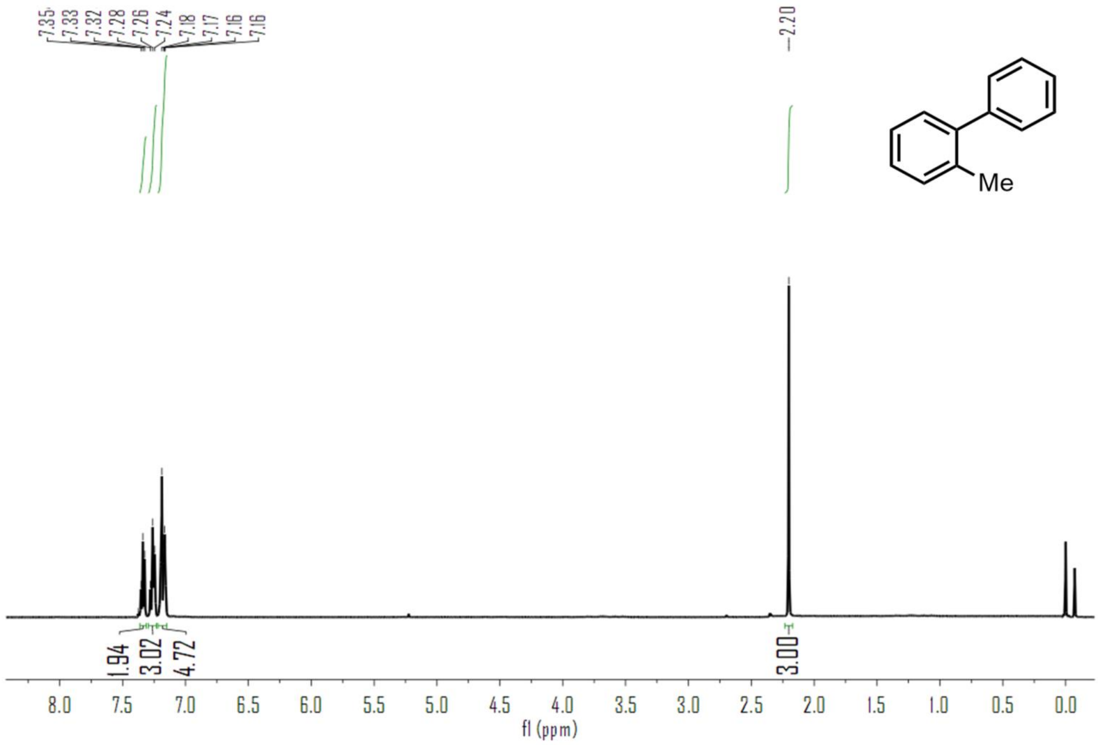

^{1}H NMR of 2-methyl-1,1'-biphenyl (3l)

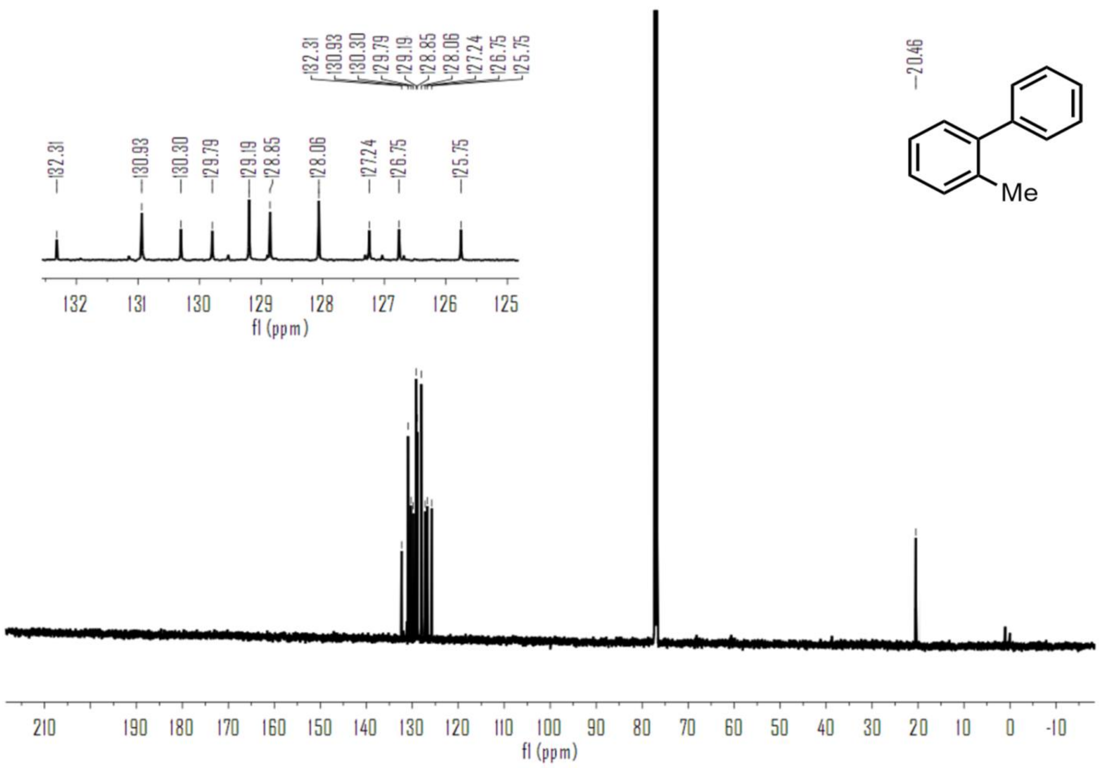

^{1}H NMR of 1-phenylnaphthalene (3m)

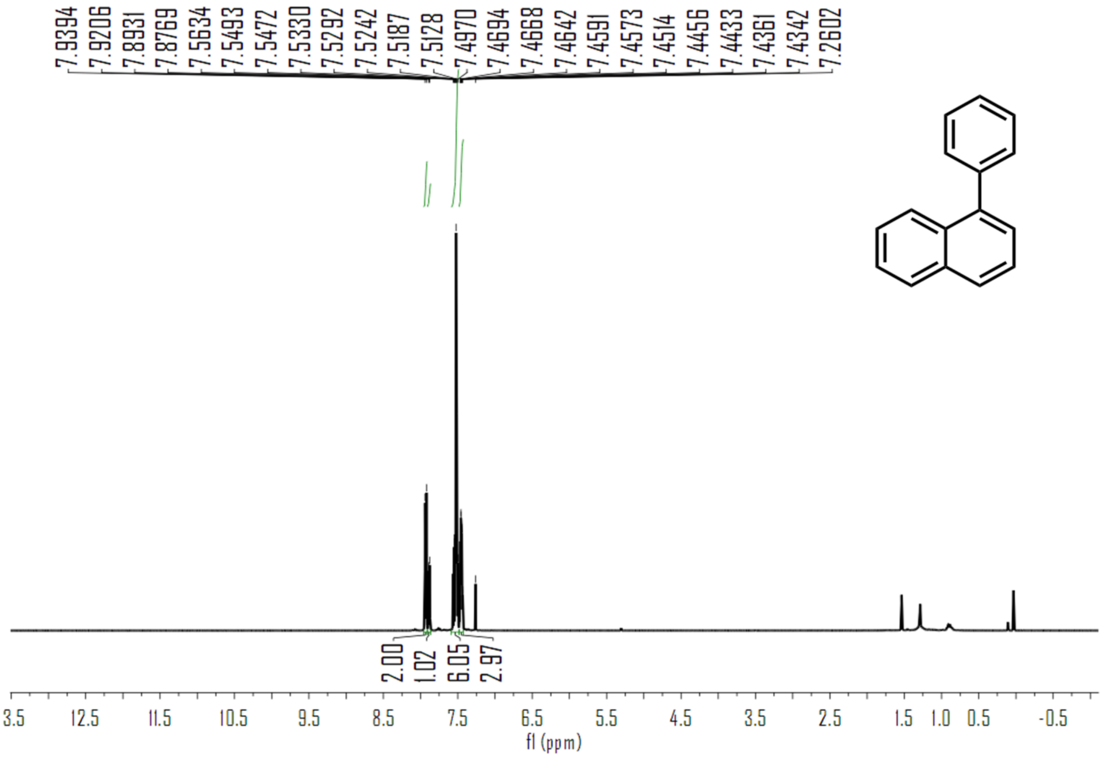

^{13}C NMR of 1-phenylnaphthalene (3m)

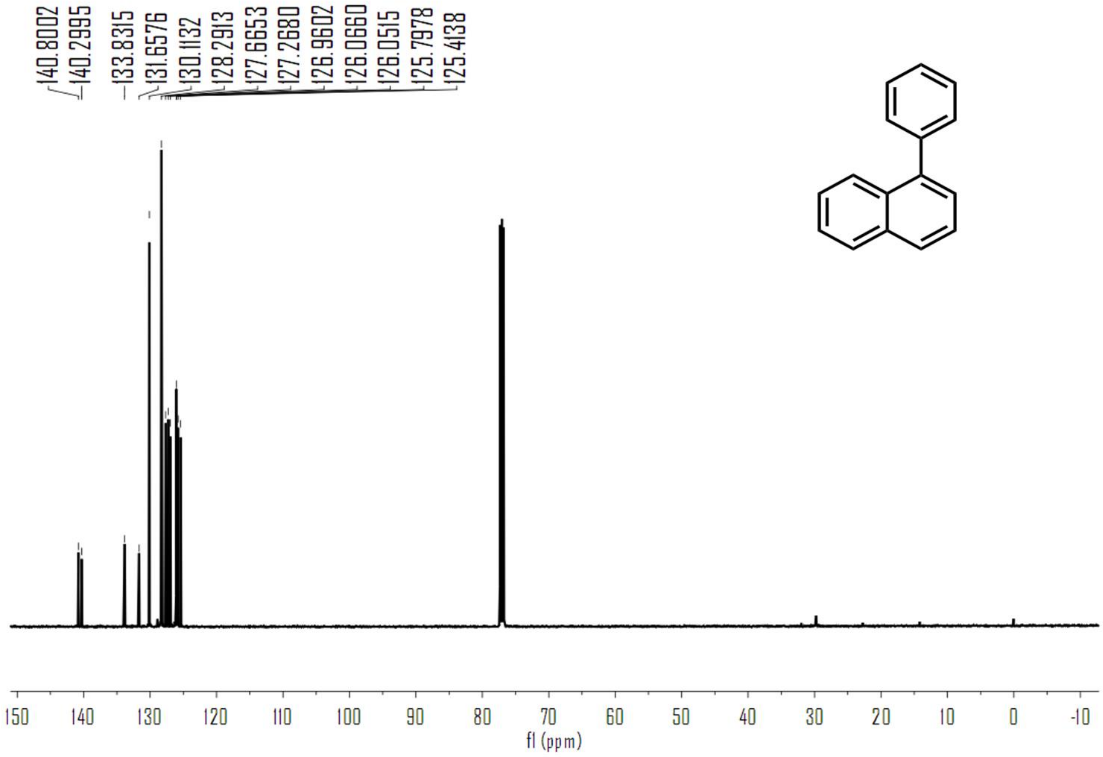

^{1}H NMR of 4-methyl-4'-(trifluoromethyl)-1,1'-biphenyl (3n)

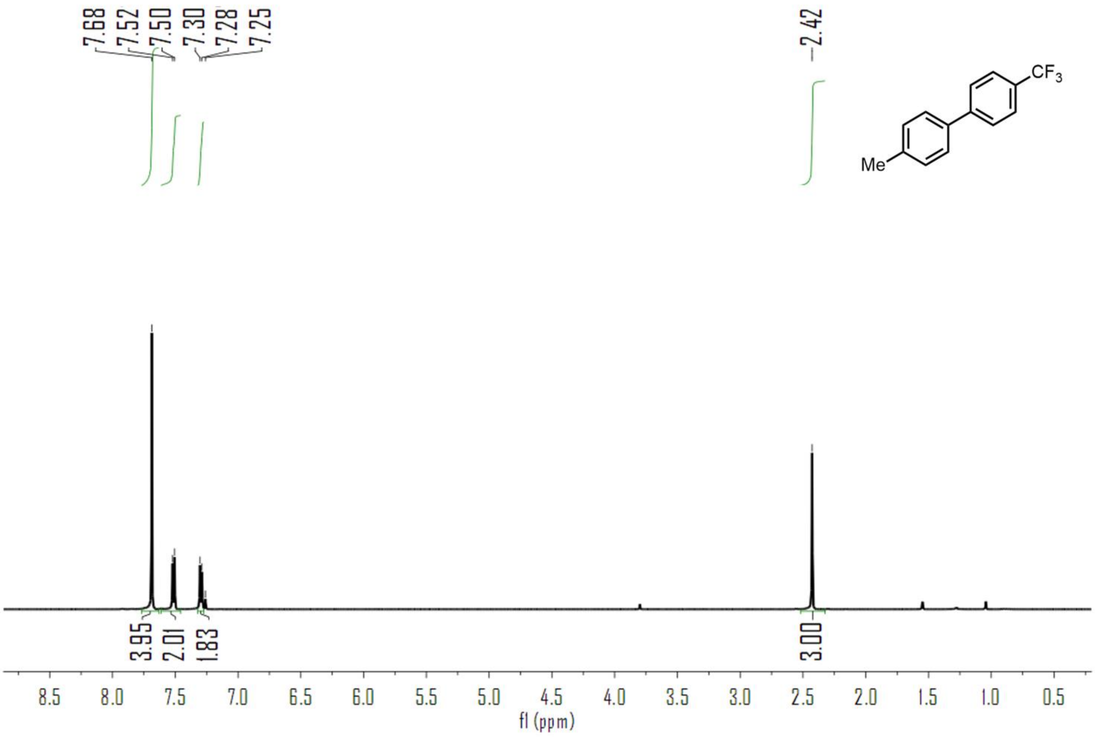

^{13}C NMR of 4-methyl-4'-(trifluoromethyl)-1,1'-biphenyl (3n)

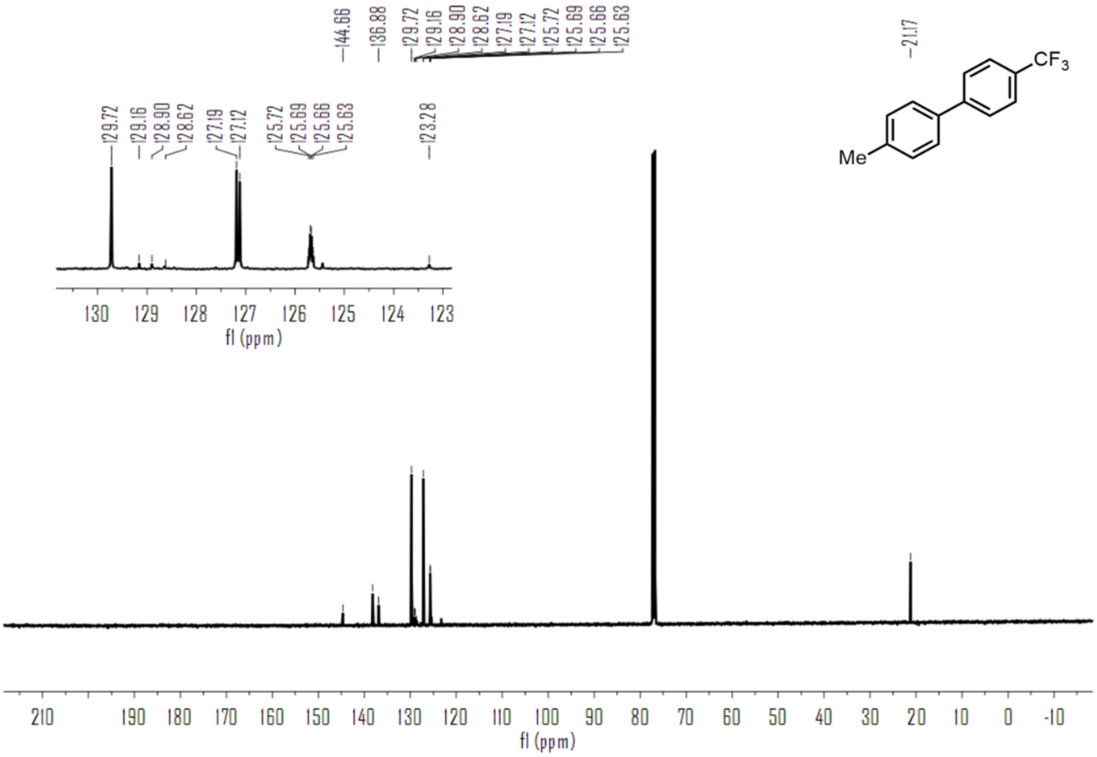

^{19}F NMR of 4-methyl-4'-(trifluoromethyl)-1,1'-biphenyl (3n)

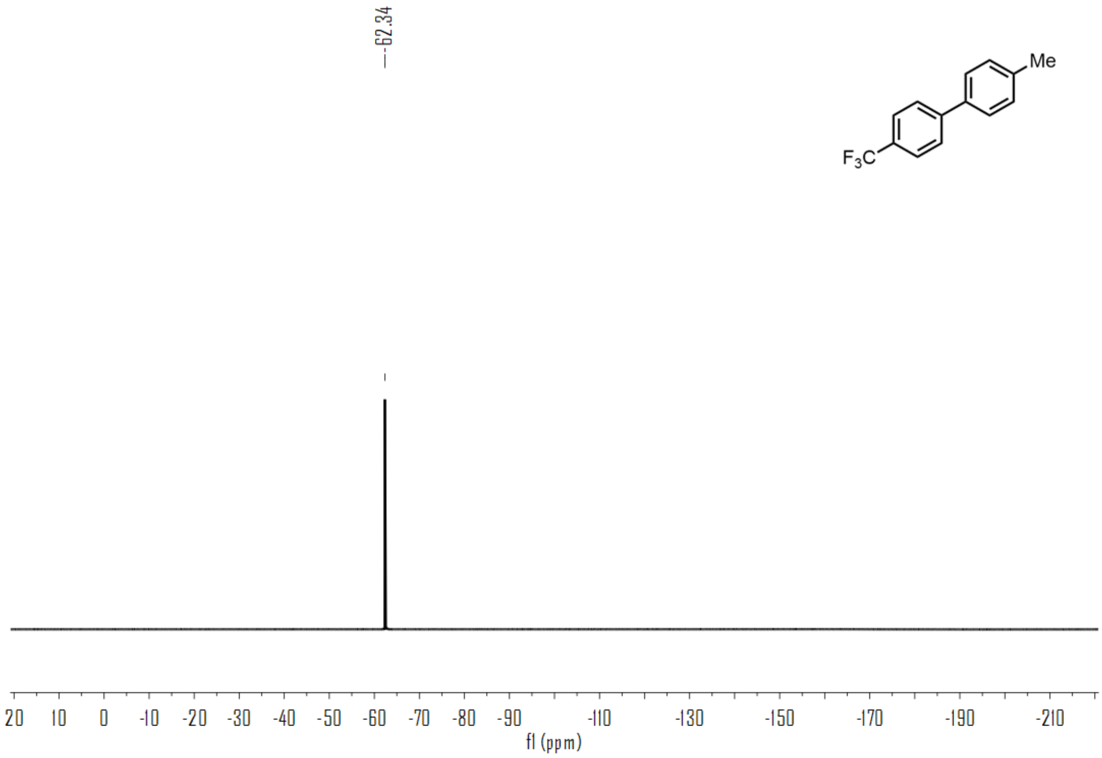

-62.34
Me
F3C
20 10 0 -10 -20 -30 -40 -50 -60 -70 -80 -90 -110 -130 -150 -170 -190 -210
f1 (ppm)

^{1}H NMR of 4'-methyl-[1,1'-biphenyl]-4-carbonitrile (3o)

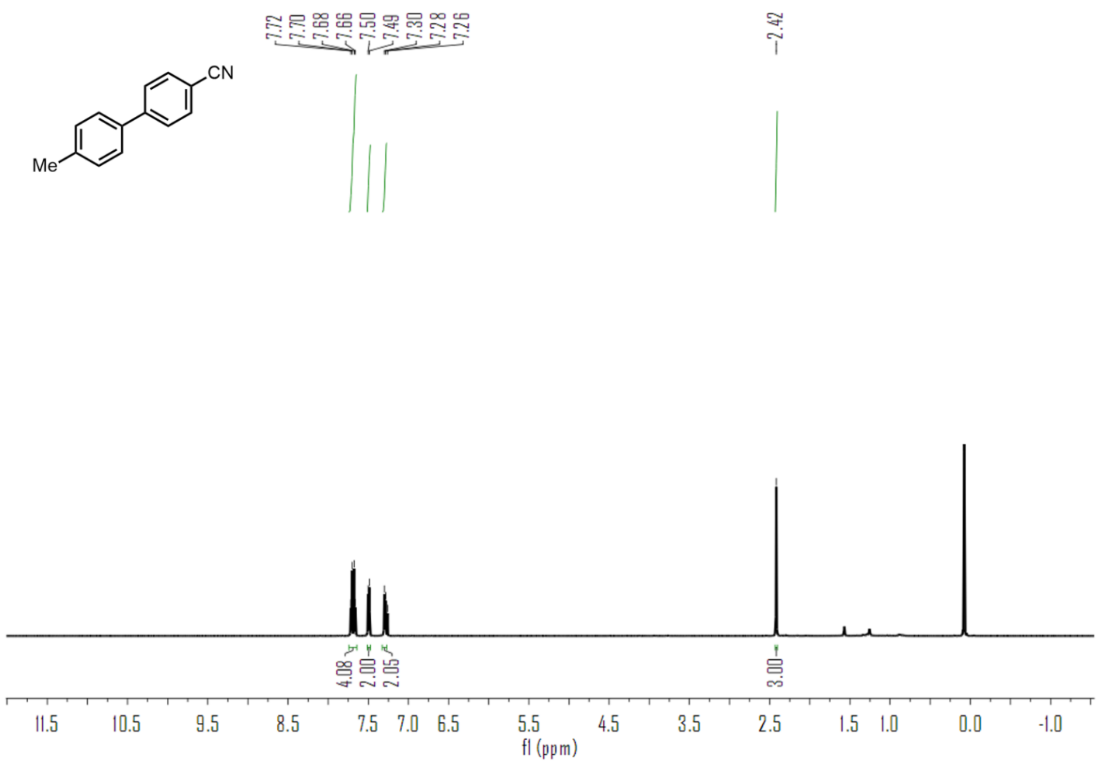

^{13}C NMR of 4'-methyl-[1,1'-biphenyl]-4-carbonitrile (3o)

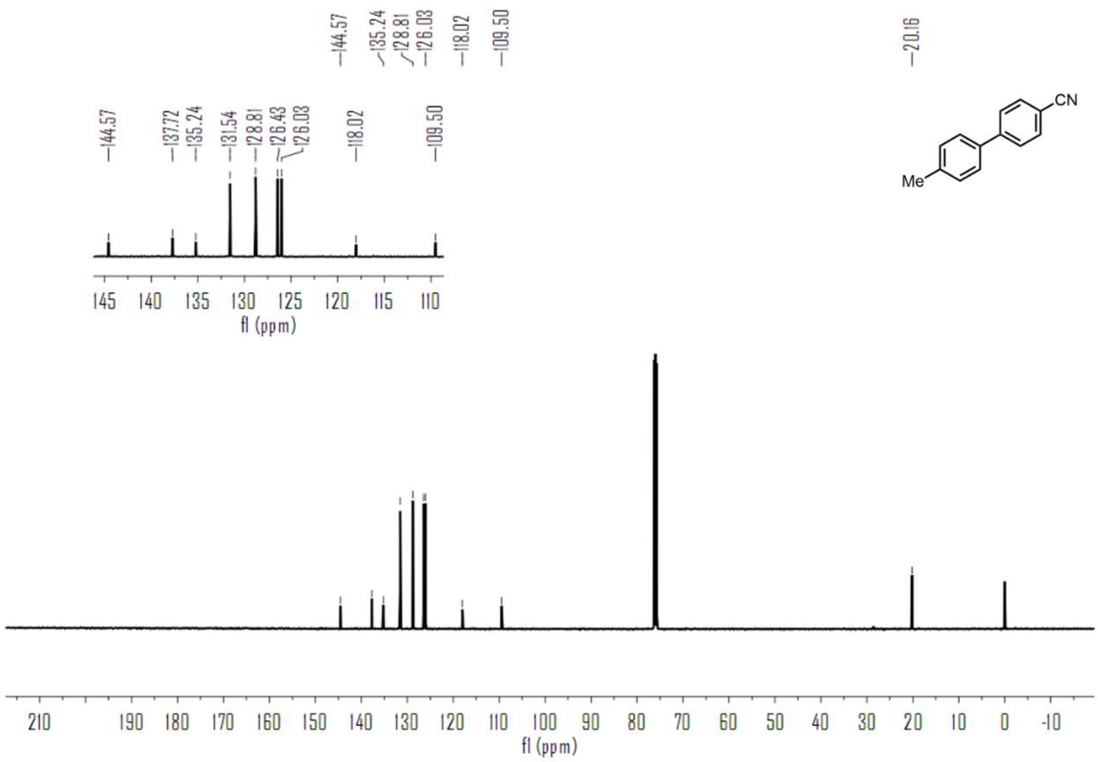

^{1}H NMR of trimethyl(4'-methyl-[1,1'-biphenyl]-4-yl)silane (3p)

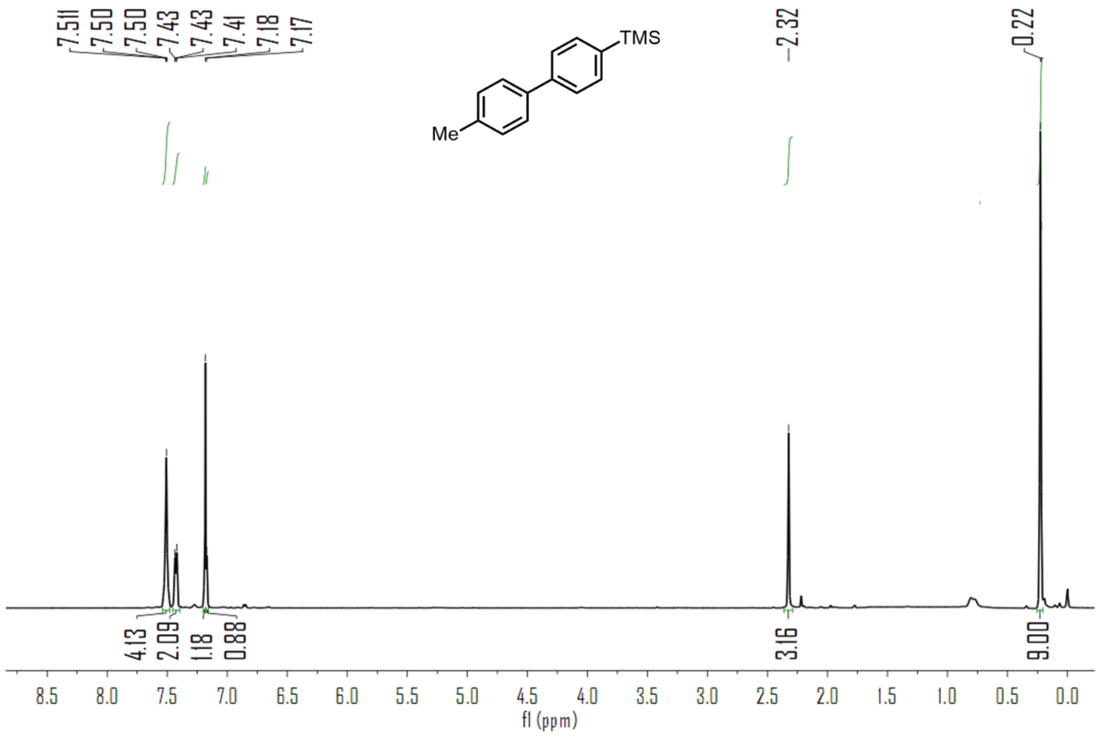

^{13}C NMR of trimethyl(4'-methyl-[1,1'-biphenyl]-4-yl)silane (3p)

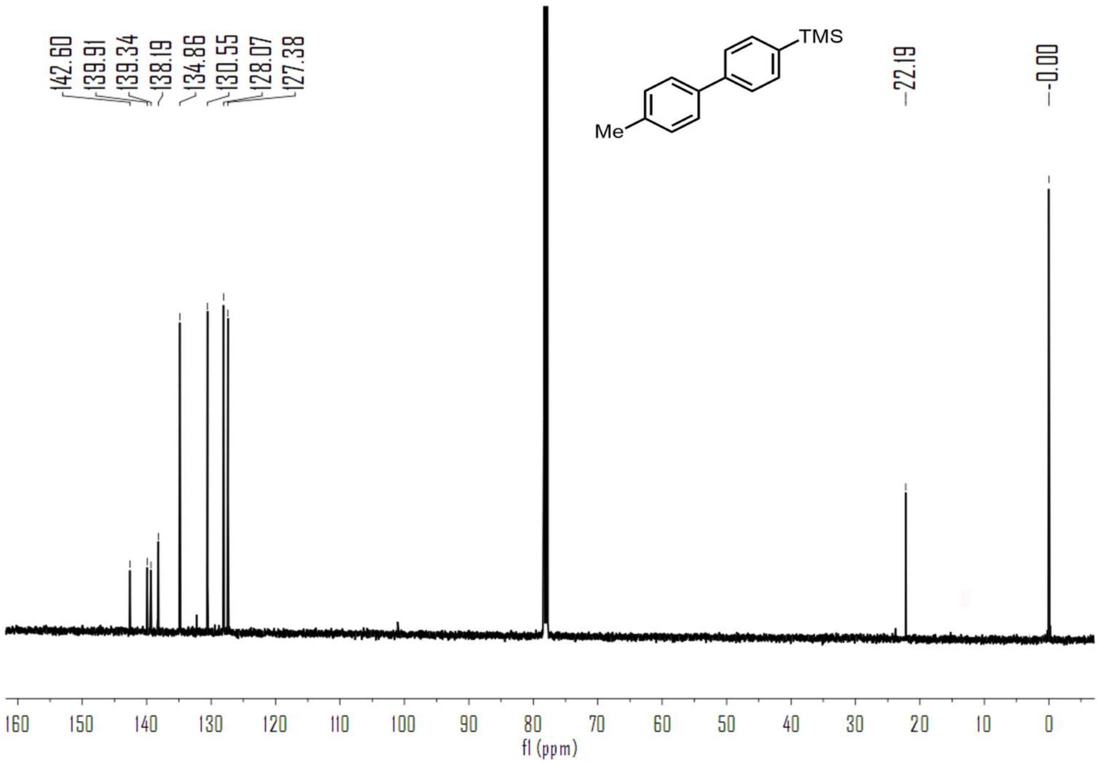

^{1}H NMR of 4,4'-dimethyl-1,1'-biphenyl (3q)

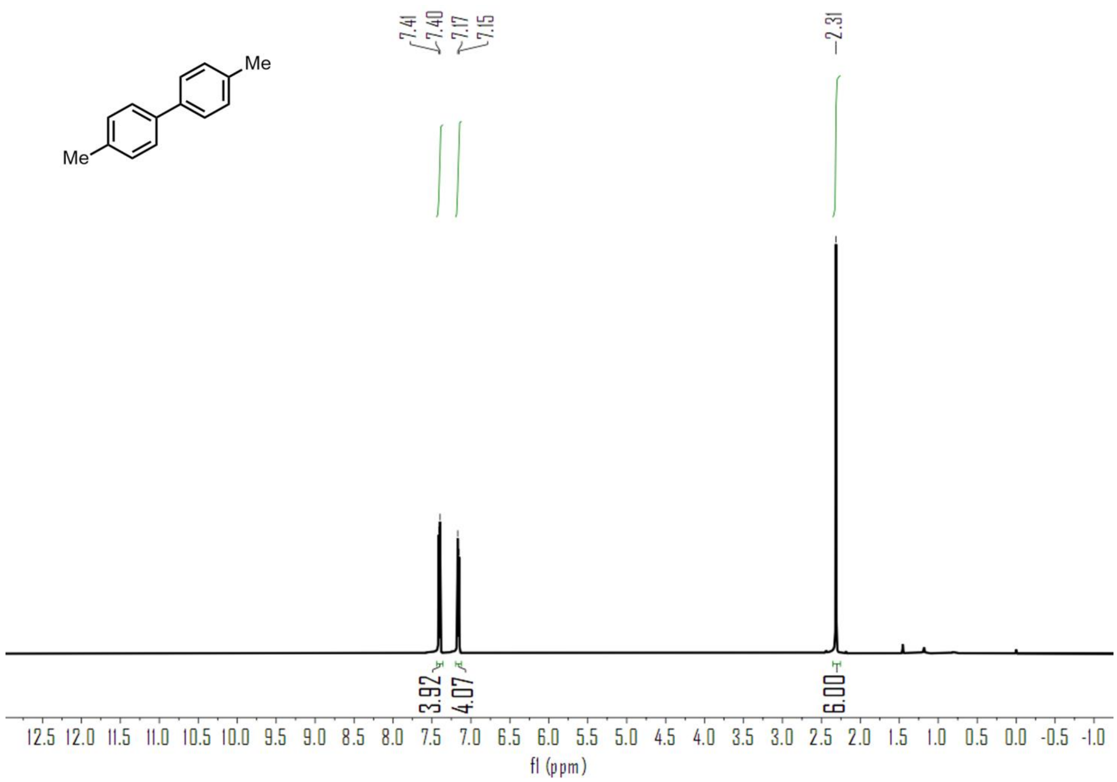

^{13}C NMR of 4,4'-dimethyl-1,1'-biphenyl (3q)

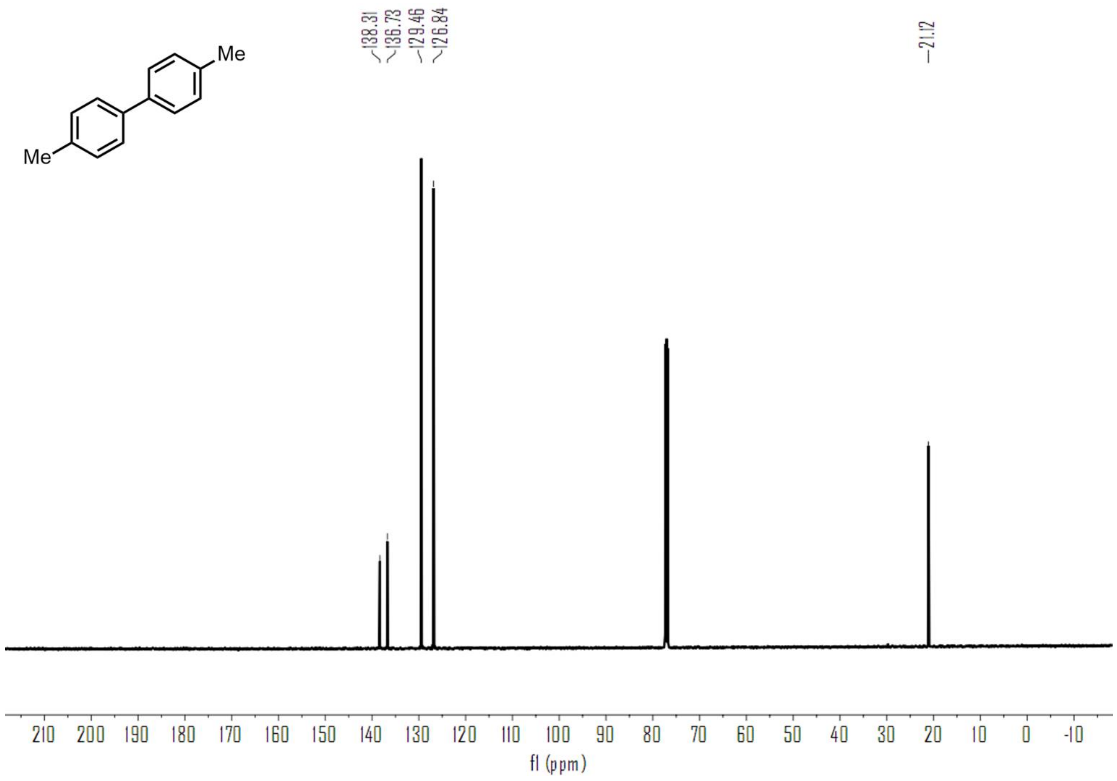

^{1}H NMR of4-methyl-4'-phenoxy-1,1'-biphenyl (3r)

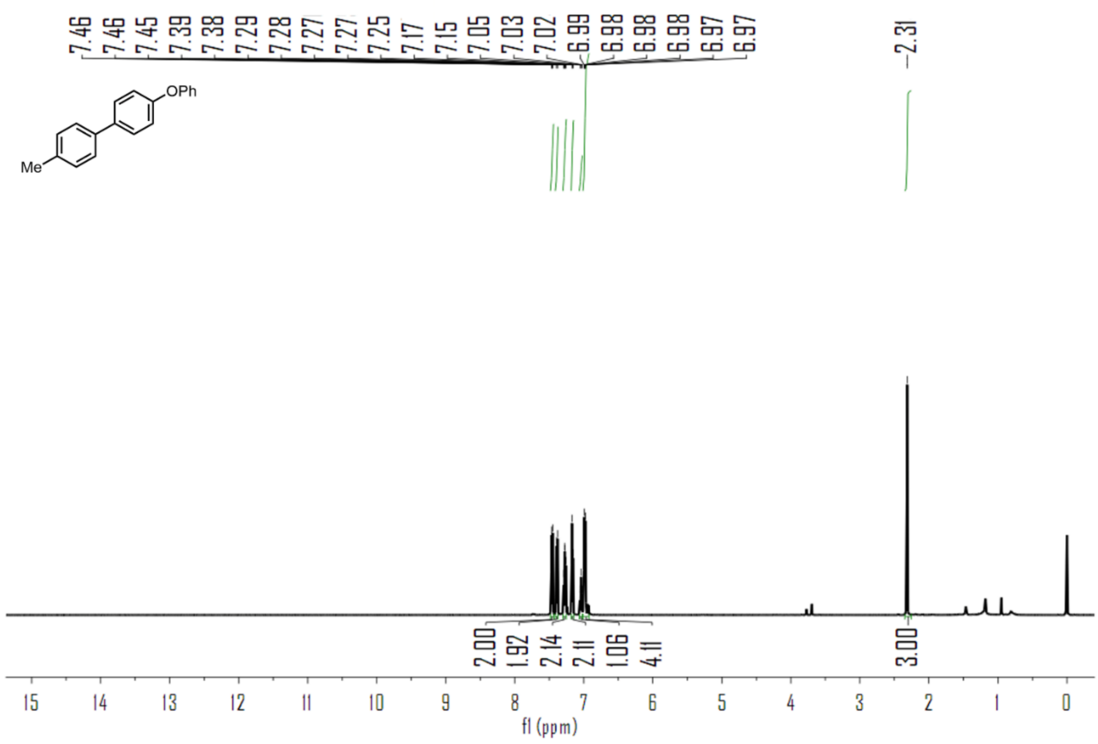

^{13}C NMR of 4-methyl-4'-phenoxy-1,1'-biphenyl (3r)

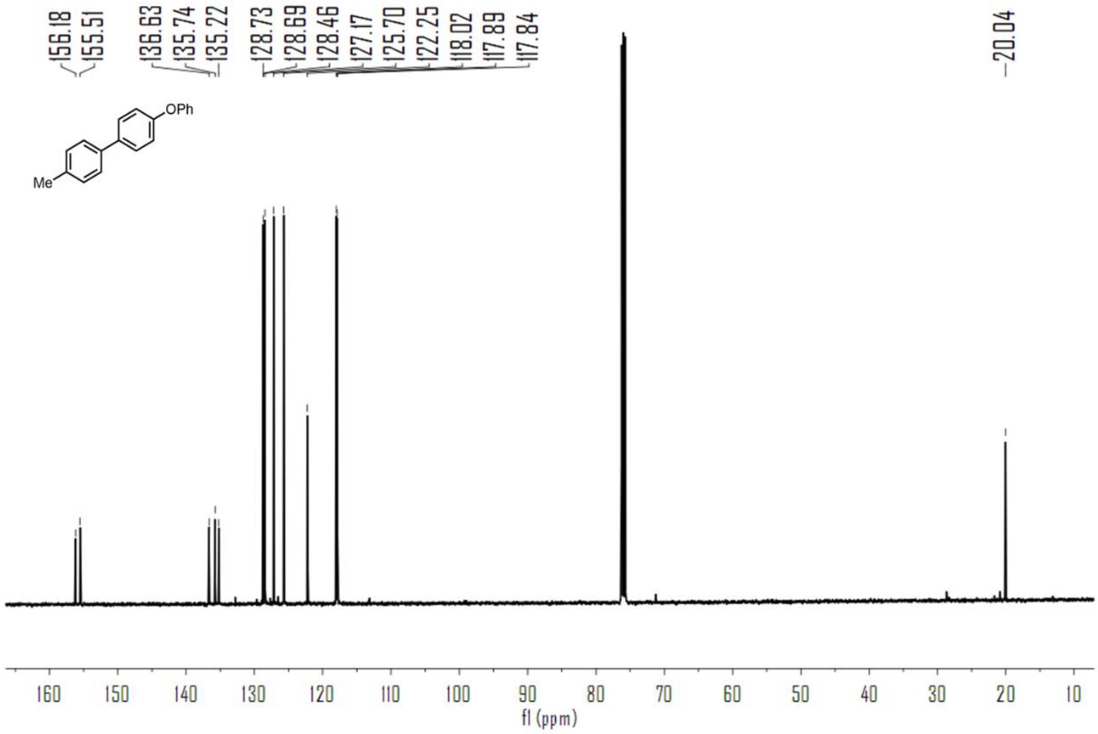

^{1}H NMR of 3-fluoro-4'-methyl-1,1'-biphenyl (3s)

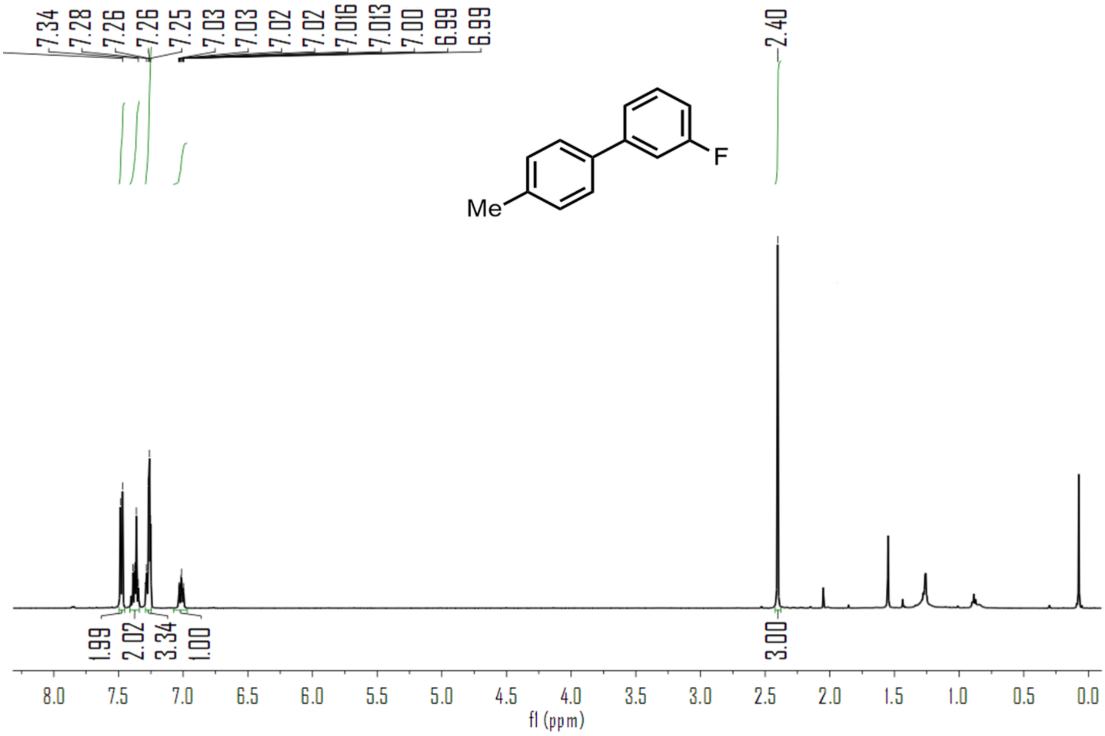

^{13}C NMR of 3-fluoro-4'-methyl-1,1'-biphenyl (3s)

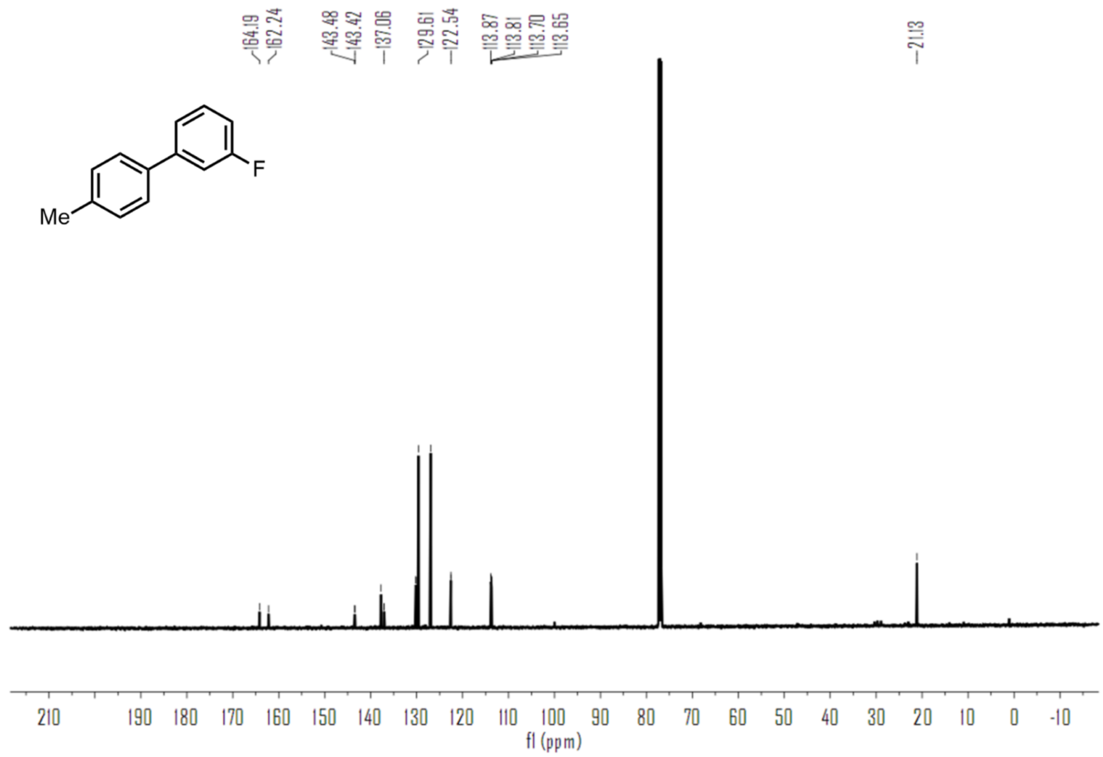

^{19}F NMR of 3-fluoro-4'-methyl-1,1'-biphenyl (3s)

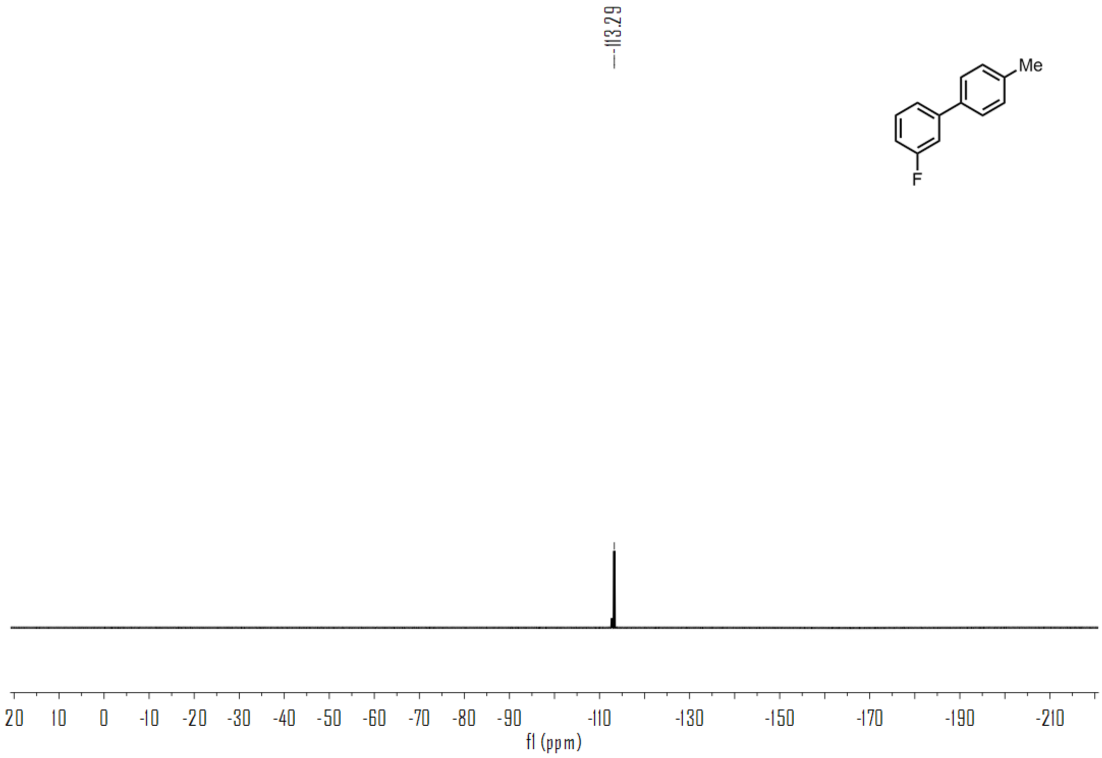

113.29
Me
F
20 10 0 -10 -20 -30 -40 -50 -60 -70 -80 -90 -110 -130 -150 -170 -190 -210
f1 (ppm)

^{1}H NMR of 4'-methyl-3-(trifluoromethyl)-1,1'-biphenyl (3t)

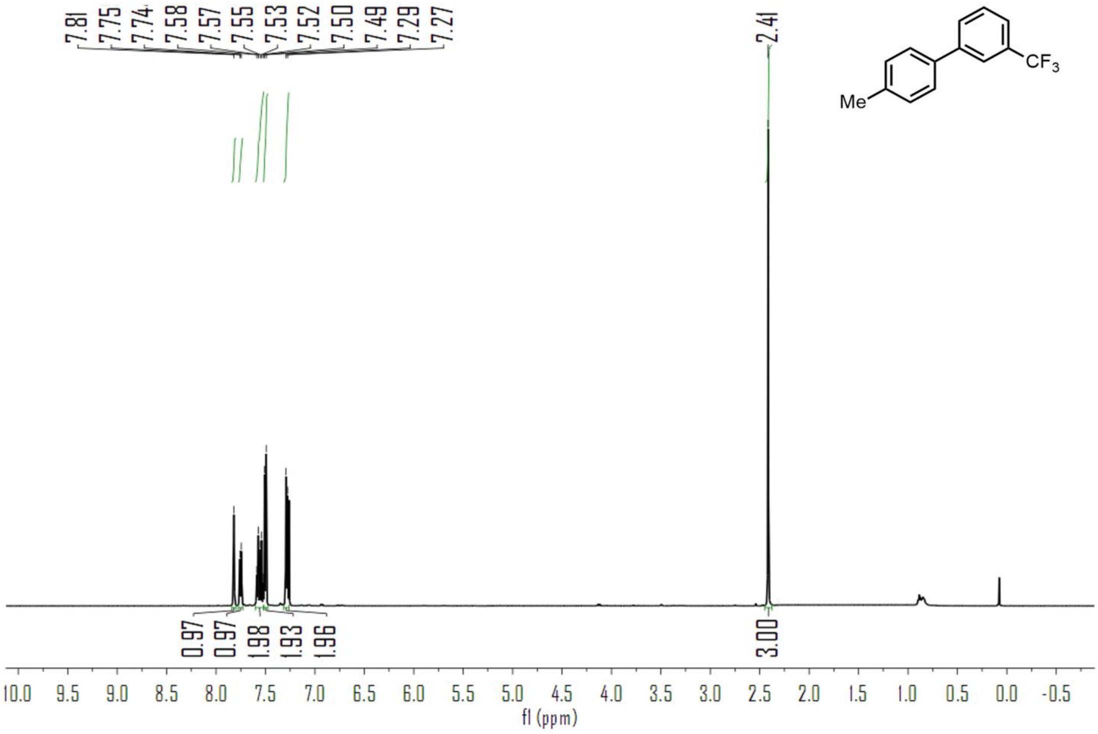

^{13}C NMR of 4'-methyl-3-(trifluoromethyl)-1,1'-biphenyl (3t)

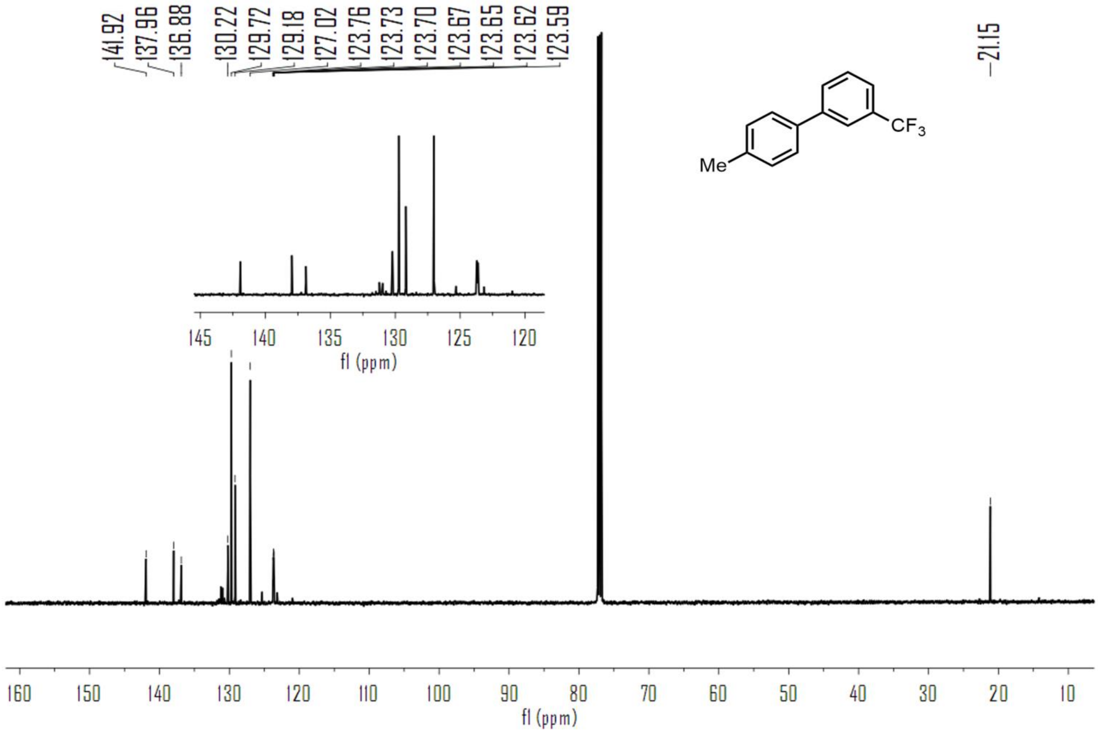

^{19}F NMR of 4'-methyl-3-(trifluoromethyl)-1,1'-biphenyl (3t)

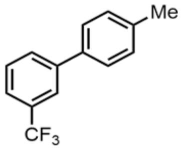
Me
CF3

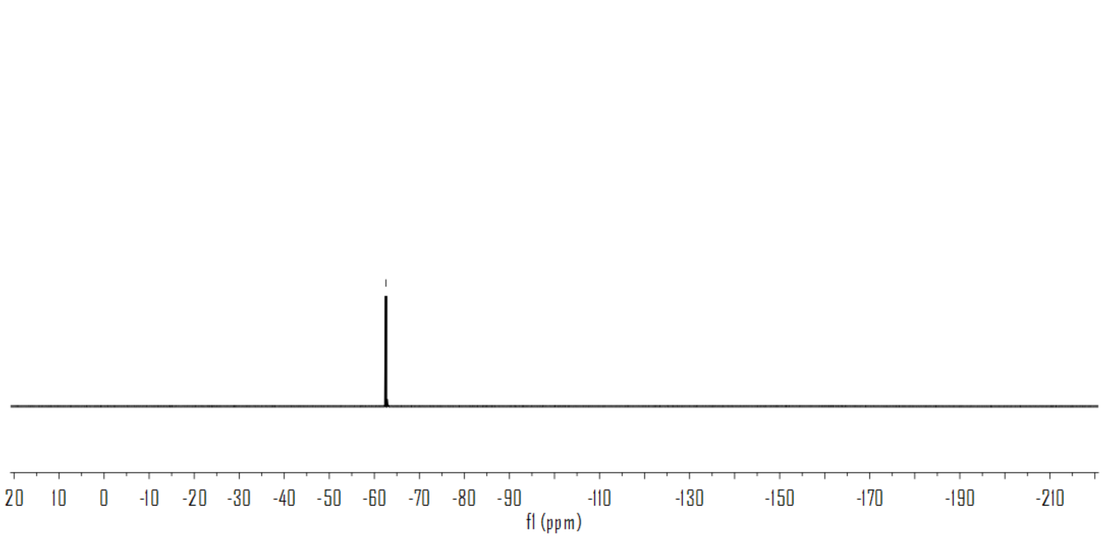
-62.59
20
10
0
-10
-20
-30
-40
-50
-60
-70
-80
-90
-110
-130
-150
-170
-190
-210
f1 (ppm)

^{1}H NMR of 4-methoxy-3,4'-dimethyl-1,1'-biphenyl (3u)

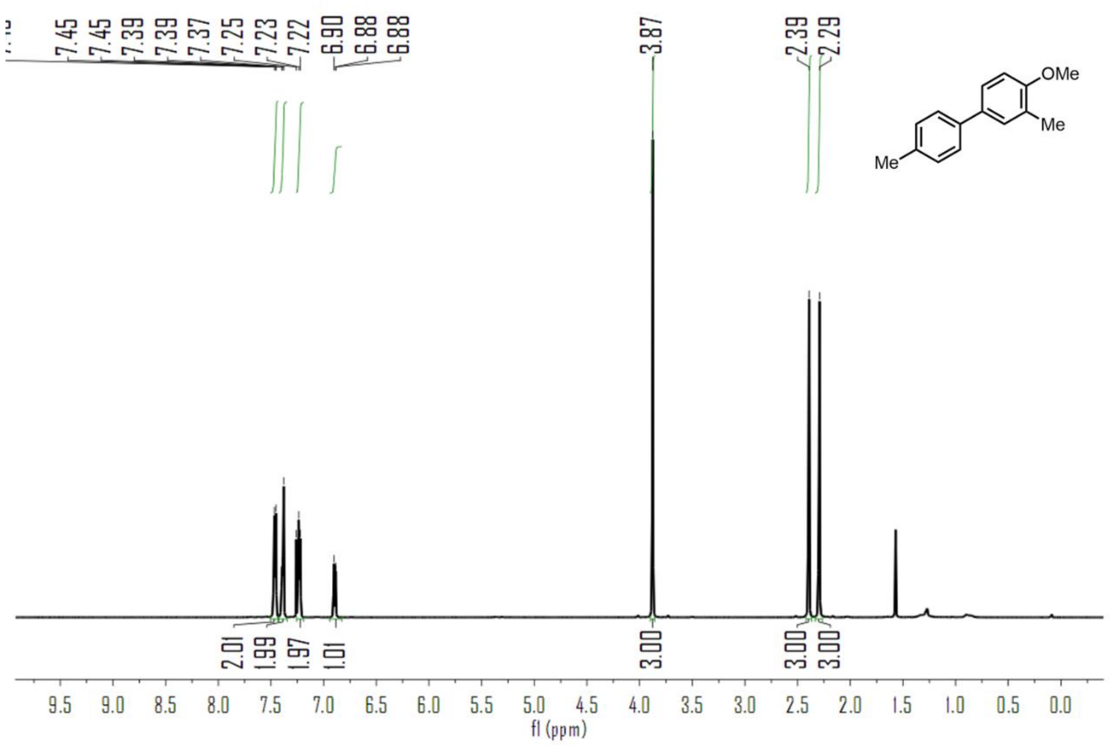

^{13}C NMR of 4-methoxy-3,4'-dimethyl-1,1'-biphenyl (3u)

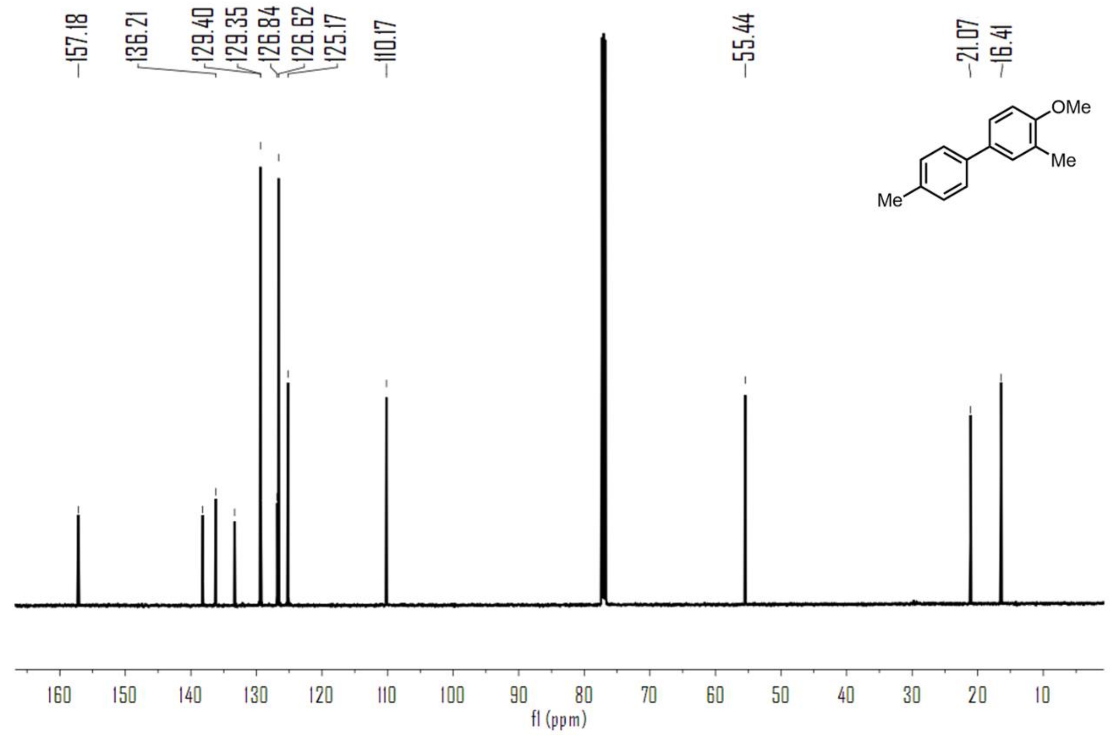

^{1}H NMR of 3,4-dimethoxy-4'-methyl-1,1'-biphenyl (3v)

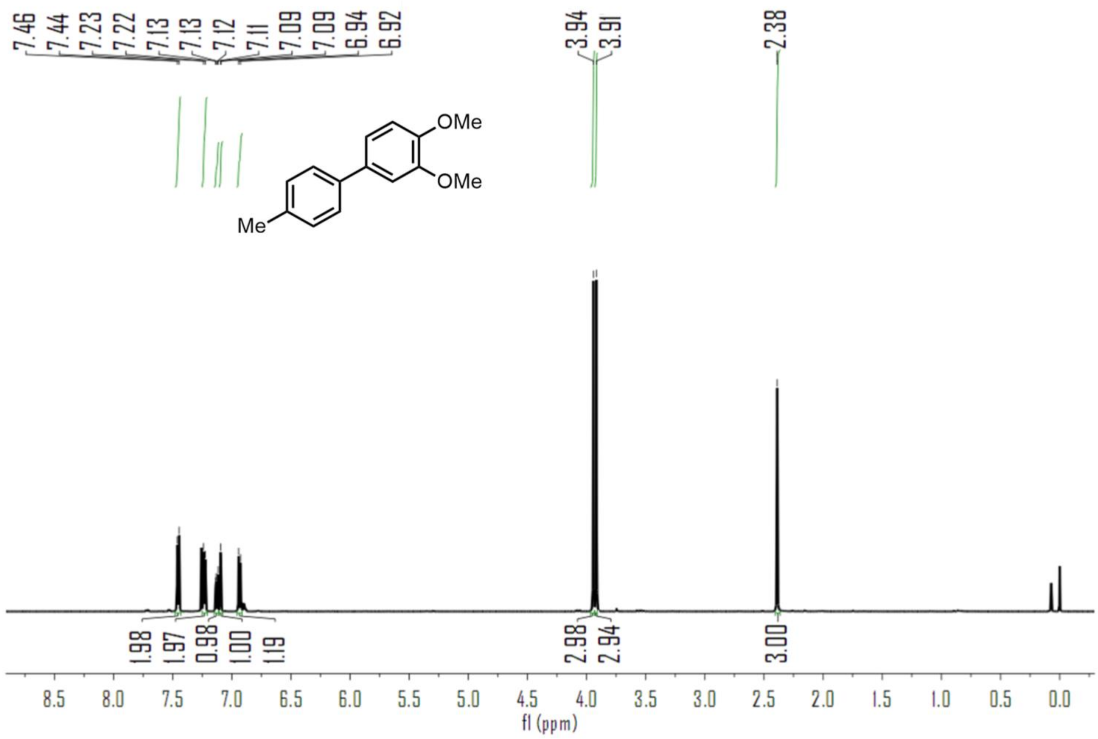

^{13}C NMR of 3,4-dimethoxy-4'-methyl-1,1'-biphenyl (3v)

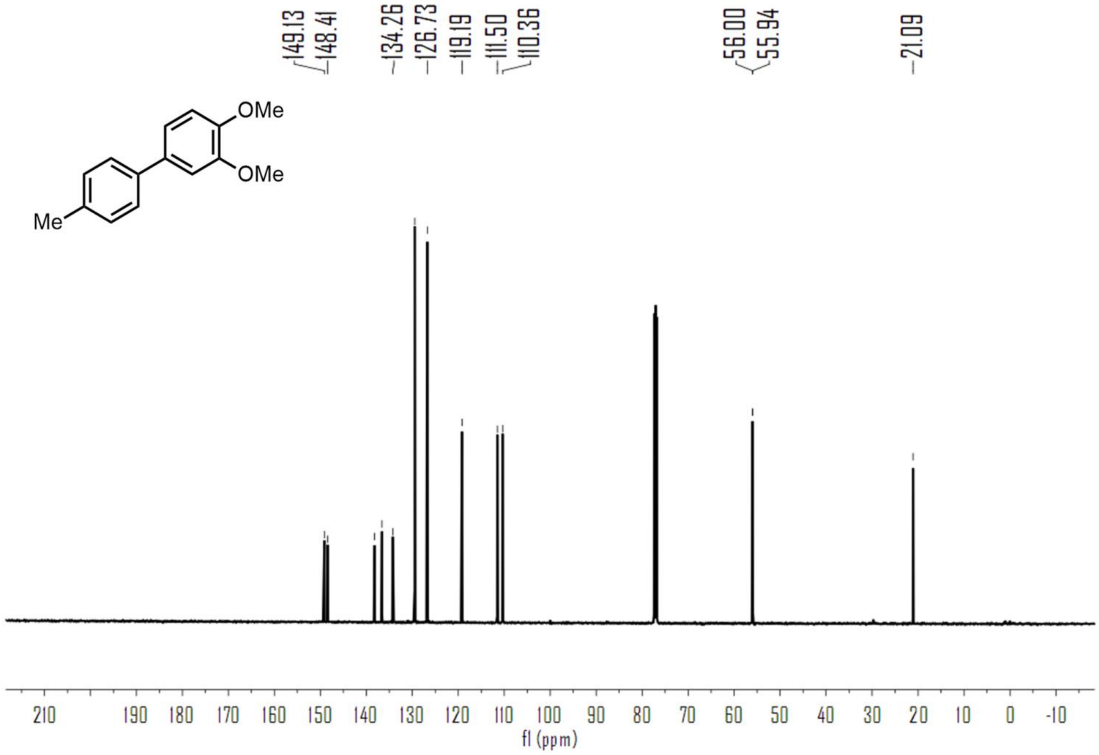

^{1}H NMR of 2-(p-tolyl)naphthalene (3w)

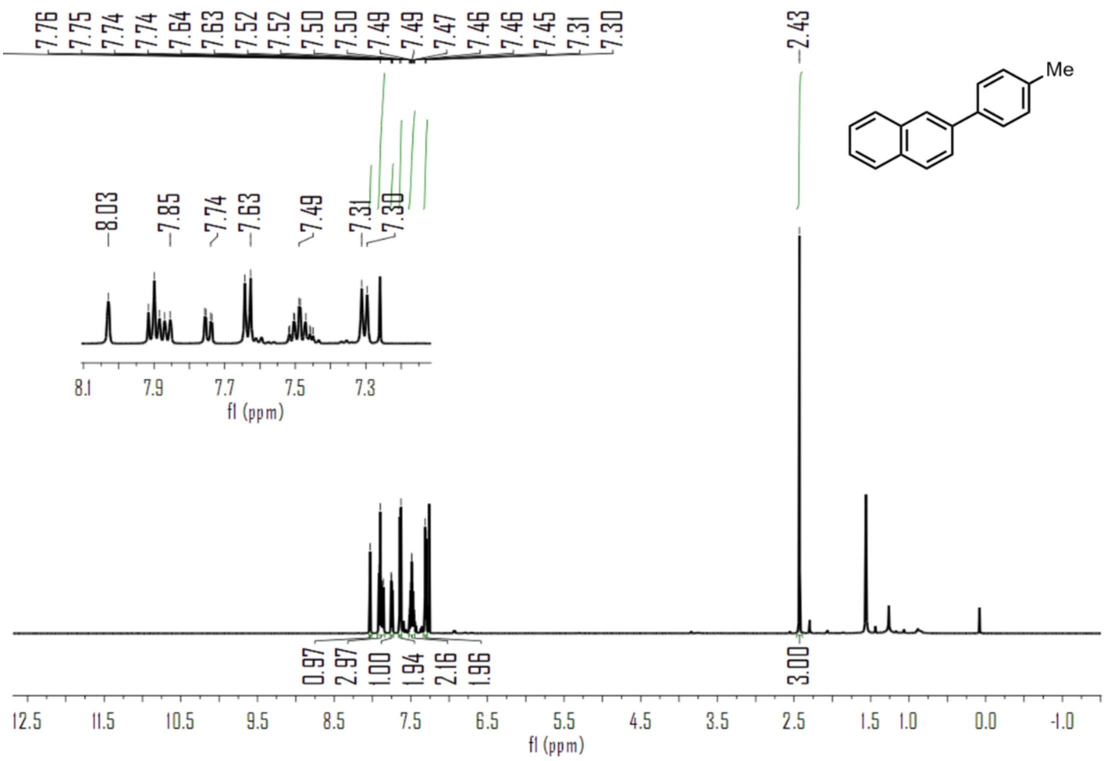

^{13}C NMR of 2-(p-tolyl)naphthalene (3w)

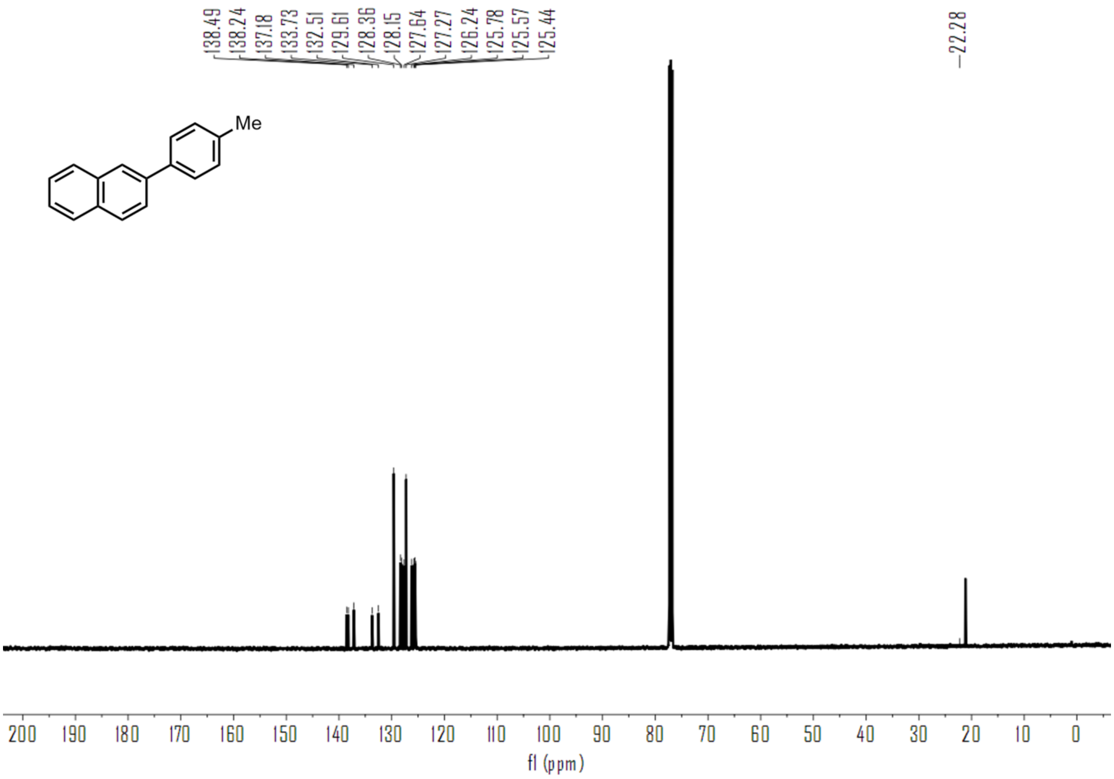

3. Data of GPC

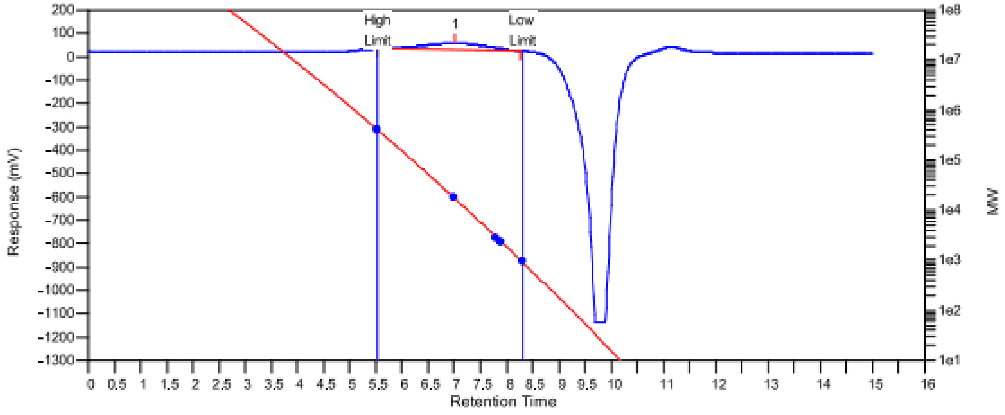

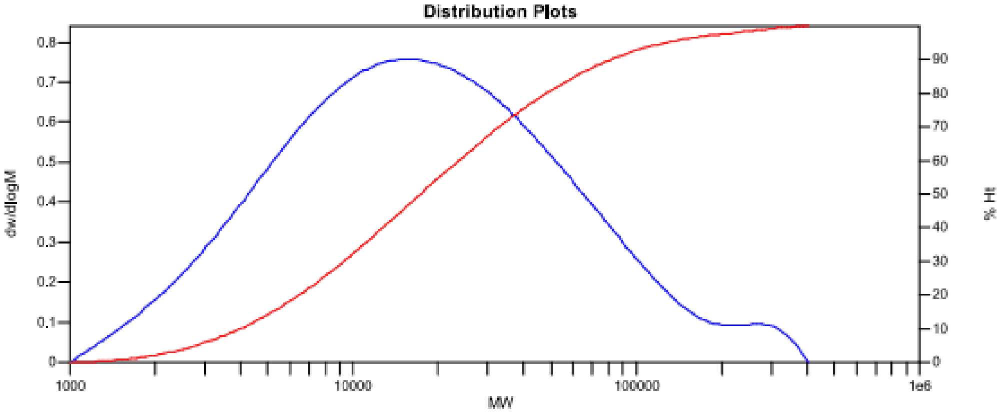

MW Averages

Peak No	Mp	Mn	Mw	Mz	Mz+1	Mv	PD
1	15170	9817	35118	108016	197531	28840	3.57726

Processed Peaks

Peak No	Name	Start RT (mins)	Max RT (mins)	End RT (mins)	Pk Height (mV)	% Height	Area (mV.secs)	% Area
1		5.53	7.02	8.27	26.9972	0	2236.5	100
